# Functional investigation of the RNA helicase MOV10 with respect to its interplay with factors involved in nonsense-mediated mRNA decay

**DOI:** 10.1016/j.jbc.2025.110418

**Published:** 2025-06-24

**Authors:** Guangpu Xue, Gabriel P. Faber, Lea S. Pommerening, Megha Mallick, Aditi Gupta, Markus C. Wahl, Yaron Shav-Tal, Sutapa Chakrabarti

**Affiliations:** 1 Institute of Chemistry and Biochemistry, Freie Universität Berlin, Berlin, Germany; 2 The Mina & Everard Goodman Faculty of Life Sciences & Institute of Nanotechnology, Bar-Ilan University, Ramat Gan, Israel; 3 Laboratory of Structural Biochemistry, Institute of Chemistry and Biochemistry Freie Universität Berlin, Berlin, Germany

**Keywords:** SF1 RNA helicase, nonsense-mediated mRNA decay, P-bodies, stress granules, protein-protein interactions

## Abstract

The RNA helicase Moloney leukemia virus 10 (MOV10) is involved in several RNA processing pathways, including RNA silencing, defense against viral RNA and nonsense-mediated mRNA decay (NMD). MOV10 is a member of the Up-frameshift 1 (UPF1)-family of superfamily 1 (SF1) helicases and like its prototype member, unwinds RNA duplexes bearing a 5′-single-stranded overhang. Sequence comparisons of MOV10 and UPF1 revealed significant identity between their RecA domains and considerable divergence between the N-terminal domains preceding the helicase core. Using *in vitro* biochemical approaches, we show that the N-terminal domain of MOV10 is functionally distinct from the CH domain of UPF1, both in terms of its impact on catalytic activity and the protein-protein interactions it mediates. MOV10 engages the NMD factor UPF2 *via* its N-terminal regulatory domain but binds a different region than the UPF1-CH domain. We propose that the interactions mediated by the MOV10-N-terminal domain dictate its localization to cytoplasmic RNA condensates such as P-bodies and stress granules. This is distinct from UPF1, whose localization appears to be driven by its interaction with RNA. Taken together, our work presents a mechanistic model for the recruitment and involvement of MOV10 in NMD, where it was proposed to act as an RNA clearance factor for UPF1.

The dynamic regulation of gene expression in cells involves a continuous change in flux of mRNA transcripts through different RNA processing pathways. This dynamic flux depends on the modulation of RNA-RNA and RNA-protein interactions, which are often brought about by RNA-binding proteins (1). A class of proteins that play an important role in such remodeling events are RNA helicases (2). These enzymes, also referred to as RNA-dependent ATPases for their ability to hydrolyze ATP upon binding RNA, are present in all domains of life and are involved in all pathways of mRNA processing. Due to their pervasive nature, RNA helicases are often essential for cell viability (3). Eukaryotic RNA helicases belong to two of the six helicase superfamilies, superfamily (SF) 1 and 2. Most RNA helicases belong to SF2, while only a few are members of SF1 (4). Prominent among the SF1 RNA helicases is the protein Up-frameshift 1 (UPF1), which is conserved from yeast to humans and is best studied in the context of the nonsense-mediated mRNA decay (NMD) pathway (5, 6). UPF1 belongs to the SF1B family of RNA helicases that translocate and unwind nucleic acids in a 5′-3′ direction (7). Examples of other SF1B RNA helicases in humans include Senataxin (SETX), which is involved in transcription termination, IGHMBP2, which is thought to play a role in ribosome biogenesis and translational control, and Moloney leukemia virus 10 (MOV10), which is implicated in diverse RNA processing pathways (8, 9, 10, 11).

The putative helicase MOV10 was first identified *via* its interaction with the Argonaute protein, Ago2, and was thought to be a component of the RNA-induced silencing complex (RISC) (12). It was also shown to associate with APOBEC3G, a member of the family of cytidine deaminases, which play an important role in host-mediated antiviral resistance (13). These observations indicated a role for MOV10 in microRNA processing and defense against exogenous viral elements. Later, studies from Gregersen et al. demonstrated ATP-dependent 5′-3′ RNA unwinding activity of MOV10, establishing it as a *bona fide* RNA helicase, and suggesting that these activities are necessary for the physiological functions (14). In addition, PAR-CLIP analysis of MOV10 showed that a substantial number of MOV10 mRNA targets are also bound by UPF1, implying that MOV10 might play a role in UPF1-mediated decay pathways. Correspondingly, depletion of MOV10 stabilized several NMD target transcripts as well as a β-globin NMD reporter transcript.

Although the NMD pathway involves three RNA helicases, UPF1, DHX34, and MOV10, UPF1 is the only essential component and the central factor of NMD. Previous studies proposed that the function of UPF1 is to actively remodel the target messenger ribonucleoprotein particle (mRNP) to release associated proteins in preparation for degradation (15). More recently, UPF1 was shown to use its ATPase activity to scan RNA *via* rapid binding and ATPase-stimulated dissociation, to identify cognate NMD targets and thereby maintain stringency in target selection (16). DHX34 was shown to facilitate UPF1 phosphorylation by the kinase SMG1, while MOV10 was suggested to act as an RNA clearance factor for UPF1, outlining distinct roles for the three helicases in the NMD pathway (14, 17). The involvement of two SF1 helicases in decay of certain NMD targets raises the question if the mode of recruitment and regulation of the two proteins are distinct from each other. It has been shown that although MOV10 and UPF1 interact and bind close to each other on the 3′-untranslated region (UTR) of the mRNA target, the recruitment of one does not depend on the other. The interactions of UPF1 with the exon junction complex (EJC) *via* its associated proteins (UPF2 and UPF3), and regulation of UPF1 catalytic activity by intramolecular and intermolecular interactions are well characterized (18, 19, 20, 21). In contrast, very little is known about the catalytic activity and regulation of MOV10, as well as its recruitment to UPF1-mediated decay pathways. To gain insights into these aspects, we undertook a biochemical approach and used recombinant proteins to test for the catalytic and RNA-binding activities of MOV10, as well as its interaction with core NMD factors. We show that the primary difference between MOV10 and UPF1 lies in their N-terminal regulatory domains, which mediate distinct intermolecular and intramolecular interactions, leading to differences in catalytic activity and regulation of the two helicases. Furthermore, using fluorescence microscopy, we show that MOV10 and UPF1 exhibit distinct patterns of localization to cytoplasmic RNA-rich bodies, suggesting a functional divergence that likely arises from different protein-protein/RNA interactions mediated by the two SF1 helicases.

## Results

### The distinct N-terminal domains of MOV10 and UPF1 mediate different effects on their RNA-binding and catalytic activities

**Figure 1:**
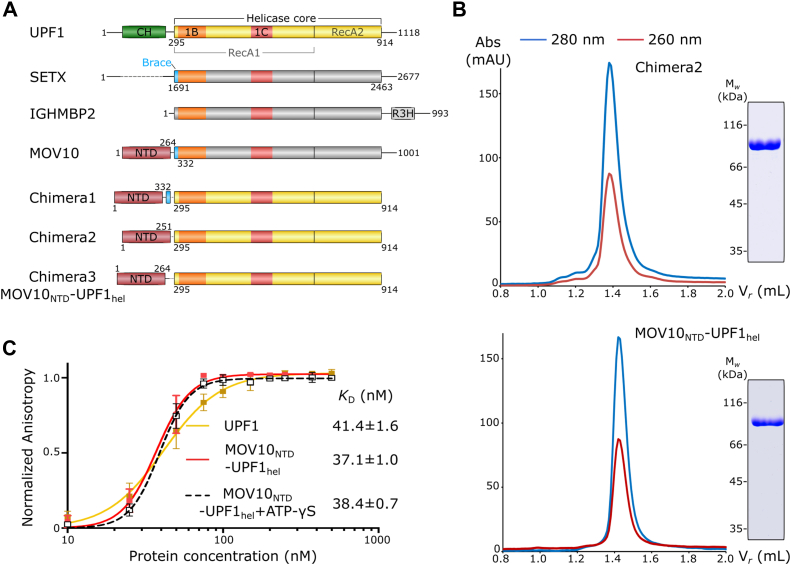
**A functional chimera of the MOV10 N-terminal domain and UPF1 helicase core.***A*, schematic representation of the domain organization of SF1 RNA helicases: UPF1, SETX, IGHMBP2, and MOV10. The conserved core of SF1 RNA helicases comprises the two RecA domains (colored *yellow* in UPF1 and *gray* in the other superfamily members) and domains 1B and 1C (*orange* and *red*, respectively) that are embedded in the RecA1 domain. Certain superfamily members contain additional auxiliary domains/regions flanking the helicase core, such as the CH domain of UPF1 (*green*) and the N-terminal domain (NTD) of MOV10 (*brown*). Chimeras 1, 2 and 3 denote the three variants of the MOV10-UPF1 fusion polypeptide generated by linking the MOV10-NTD with the helicase core of UPF1. *B*, analytical size-exclusion chromatography and corresponding SDS-PAGE analyses of purified Chimera 2 and 3. All further biochemical experiments were performed with Chimera 3, hereafter, referred to as MOV10_NTD_-UPF1_hel_. *C*, quantification of RNA-binding affinities of MOV10_NTD_-UPF1_hel_, in the absence (*red trace*) and presence of the nonhydrolyzable ATP analogue, ATPγS, (*black dashed trace*) using fluorescence anisotropy. The data points and their associated error bars represent the mean and standard deviation of three independent measurements. The data were fit to a one-site binding model to determine the dissociation constant (*K*_D_, reported as the mean and standard error of mean) in each case. RNA binding of UPF1 in the absence of nucleotide (*yellow*) is shown for comparison. MOV10_NTD_-UPF1_hel_ binds RNA with an affinity comparable to that of UPF1 but unlike UPF1, does not show a decrease in affinity upon addition of an ATP analogue (see Fig. S3*C*). MOV10, Moloney leukemia virus 10; SF1, superfamily 1; UPF1, Up-frameshift 1.

Our first step towards understanding the mechanisms of MOV10 activity was to compare the domain organization of the four human SF1B RNA helicases, namely the prototype UPF1, SETX, IGHMBP2 and MOV10. These helicases have a conserved helicase core architecture comprising the two RecA domains, present in all helicases, and two auxiliary domains (domains 1B and 1C) embedded in the RecA1 domain that are unique to the SF1B family (Fig. 1*A*) (22, 23, 24). In contrast to the helicase core, the N- and C-terminal regions flanking the conserved core are divergent in length and sequence. IGHMBP2 lacks an N terminal extension altogether and has a structured R3H domain C terminal to the helicase core, while SETX and UPF1 have extensive N terminal extensions and comparatively small C terminal extensions. Although the N terminal extensions of SETX and UPF1 bear no structural or sequence similarity, they both fold back upon the helicase core to inhibit catalytic activity, indicating a convergence in function (18, 25). The N terminus of MOV10 was predicted to be a cysteine-histidine rich (CH) domain like that of UPF1 but lacks the distinct sequence motifs that make up the zinc-finger domains (Fig. S1*A*). Comparison of the Alphafold 2-predicted structural model of full-length MOV10 with that of the experimentally determined structure of yeast UPF1 suggests that the N terminus of MOV10 adopts a very different fold from that of the UPF1 CH domain, and is organized into three distinct modules, one of which appears to be similar to the “brace” domain of yeast Senataxin, Sen1p (Fig. S1, *B* and *C*). In contrast, superposition of the helicase core domains of MOV10 (from the Alphafold 2 predicted model) and UPF1 (apo-UPF1 helicase) show significant structural similarity (Fig. S1*D*). Given the differences in sequences and structures of the N termini of the helicases, we hypothesized that any functional divergence between MOV10 and UPF1 is likely to stem from effects mediated by their distinct N-terminal domains.

To investigate the biochemical functions of MOV10 *in vitro*, we set out to recombinantly produce and purify full-length MOV10 on a large scale. Purification of full-length MOV10 from *Escherichia coli* was unsuccessful due to the insolubility of the recombinant protein. This behavior of MOV10 could not be rescued by varying expression conditions and cell strains or by fusion with soluble proteins such as thioredoxin (Trx), glutathione-S-transferase (GST), or maltose-binding protein (MBP) (Table S1). Attempts to produce full-length MOV10 using a baculovirus expression system also failed to consistently yield large amounts of homogenous protein, free of nucleic acid contaminants, that are necessary for biochemical characterization of an RNA-binding protein (Fig. S1*E*). To circumvent the problem of producing MOV10 in soluble form, we designed chimeric helicase constructs, in which the N-terminal regulatory domain of MOV10 was fused to the helicase core of UPF1 (residues 295–914) (Fig. 1*A*). Given the conservation of all functional motifs across MOV10 and UPF1, we used the UPF1 helicase core, *in lieu* of the MOV10 helicase core, for testing the impact of protein-protein interactions mediated by the MOV10 N-terminus on catalytic activity (Fig. S2).

The construct containing the complete MOV10 N-terminus, including the brace region, failed to express (Fig. S3*A*), but variants lacking the brace and including only the N-terminal domain (NTD) were soluble and could be purified to homogeneity, comparable to UPF1 (Figs. 1*B* and S1*F*). Fusions of the UPF1 CH domain (residues 115–272) with the MOV10 helicase core (including and lacking the brace) were insoluble, precluding functional analysis of the MOV10 helicase core (refer to Table S1). As the two fusion proteins containing the MOV10 NTD differed only by 13 amino acid residues, we used the variant containing the longer NTD (hereafter referred to as MOV10_NTD_-UPF1_hel_) for further biochemical studies. Comparison of the Alphafold-predicted structures of full-length MOV10 and MOV10_NTD_-UPF1_hel_ showed that the fold of the two MOV10-NTD modules is identical in both cases and is predicted with very high confidence (indicated by pLDDT scores). The relative orientation of the two NTD modules relative to each other as well as of the NTD with respect to the helicase core vary in the two predicted models, likely due to the unstructured flexible linkers (predicted with low confidence) connecting them (Fig. S3*B*).

As a first step toward analyzing MOV10_NTD_-UPF1_hel_, we determined the affinity of the fusion protein for RNA. Fluorescence anisotropy (FA) measurements using a 12-mer polyuridyl RNA (U_12_) labeled with 6-FAM at its 5′-end resulted in a dissociation constant (*K*_D_) of 36 nM for MOV10_NTD_-UPF1_hel_, comparable to that of UPF1 and UPF1_hel_ [Fig. 1*C* and (26)]. This indicates that fusion of the MOV10 NTD to the UPF1 helicase core does not distort the structure of the helicase core or affect its RNA-binding ability. It was previously reported that addition of ATP (or a nonhydrolyzable ATP analog) leads to a decrease in the RNA binding affinity of the predominant UPF1 isoform, UPF1_2_ (18, 19). This is attributed to the regulatory loop in the auxiliary domain 1B, which partially occludes the RNA binding pocket in the presence of ATP. The presence of the CH domain does not influence the behavior of the regulatory loop as this effect is observed in UPF1 variants including and lacking the CH domain (26). In contrast to UPF1, addition of the nonhydrolyzable ATP analog, ATPγS, does not lead to a decrease in RNA binding affinity of MOV10_NTD_-UPF1_hel_ (Figs. 1*C* and S3*C*). This suggests that the MOV10 NTD impacts the position of domain 1B such that the regulatory loop no longer interferes with RNA binding in presence of ATP. This is the first indication that interactions mediated by the MOV10 N terminus might differ from those of the UPF1 CH domain.

**Figure 2:**
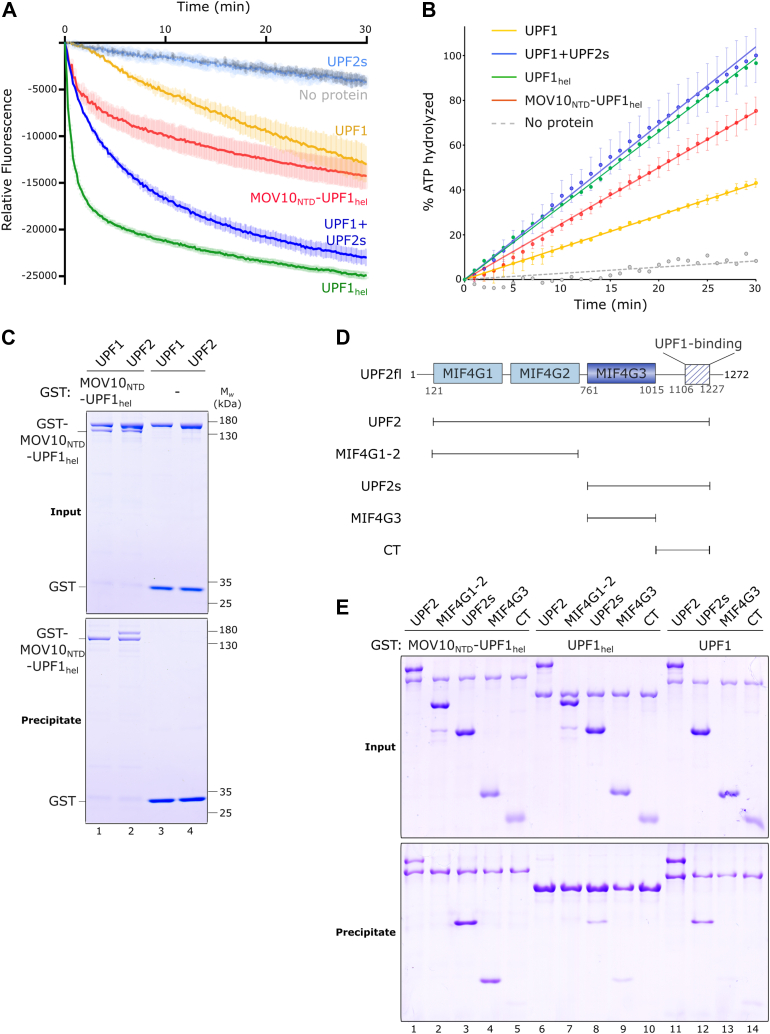
**Intramolecular and intermolecular interactions mediated by the N-terminal domain of MOV10**. *A*, fluorescence-based assay monitoring the nucleic acid-unwinding activity of MOV10_NTD_-UPF1_hel_ (*red trace*) in comparison to that of UPF1 and UPF1_hel_ (*yellow* and *green traces*, respectively). Data were recorded at 10 s intervals for 30 min. The data are displayed as the mean (*dark trace*) and standard deviation (*shaded area*) of three independent experiments, with technical duplicates in each case. The stability of the nucleic acid hybrid over the duration of the experiment is monitored by the “no protein” control. The unwinding activity of MOV10_NTD_-UPF1_hel_ is higher than the basal unwinding activity of UPF1 but substantially lower than that of UPF1_hel_ or UPF2-bound UPF1 (see also Fig. S3*D*). *B*, measurement of the RNA-dependent ATPase activity of MOV10_NTD_-UPF1_hel_ recapitulates the trend observed above for the nucleic acid unwinding activity. The RNA-stimulated ATPase activity of MOV10_NTD_-UPF1_hel_ (*red*) is higher than the basal ATPase activity of UPF1 (*yellow*) but lower than the stimulated ATPase activity of UPF1 (in a UPF2-bound state, *blue* and UPF1_hel_, *green*). The data points and their associated error bars represent the mean and standard deviation of three independent experiments (performed in duplicate). The “no protein” control monitored the spontaneous hydrolysis of ATP over time. *C*, the interaction of MOV10_NTD_-UPF1_hel_ with core NMD factors UPF1 and UPF2 was assessed using GST pull-down assays. GST-MOV10_NTD_-UPF1_hel_ was used as a bait while GST served as a negative control. The *top* and *bottompanels* show the input and precipitate, respectively. GST-MOV10_NTD_-UPF1_hel_ binds specifically to UPF2 but not UPF1. *D*, schematic representation of the domain organization of UPF2 and the UPF2 variants used in this study. The structured MIF4G domains as well as the UPF1-binding domain at the C terminus are indicated. *E*, GST pull-down assays using the UPF2 variants in (*D*) to map the MOV10_NTD_-UPF1_hel_-interacting region of UPF2. GST-UPF1_hel_ and GST-UPF1 are included as negative and positive controls, respectively. Binding of the UPF2-MIF4G3 domain to GST-MOV10_NTD_-UPF1_hel_ but not GST-UPF1_hel_ and GST-UPF1 suggests that the interaction observed with GST-MOV10_NTD_-UPF1_hel_ is specific and is mediated by the MOV10-NTD (see also Fig. S3*E*). MOV10, Moloney leukemia virus 10; NMD, nonsense-mediated mRNA decay; UPF1, protein up-frameshift 1.

The CH domain of UPF1 has an inhibitory effect on its catalytic activity. The inhibitory effect is caused by docking of the CH domain onto the RecA2 domain of UPF1, which exerts an allosteric effect on domain 1B, resulting in clamping of 1B onto the 3′-end of the RNA and impeding translocation of UPF1. Consequently, a UPF1 variant lacking the CH domain (UPF1_hel_) shows a significant increase in catalytic activity compared to UPF1. As the NTD of MOV10 appears to diverge from the UPF1 CH domain with respect to intramolecular interactions, we set out to investigate if it similarly inhibits UPF1 catalytic activity. To this end, we monitored the ability of MOV10_NTD_-UPF1_hel_ to displace a DNA strand from a complementary RNA bearing a 5′-overhang, in an ATP-dependent manner over time. Although MOV10_NTD_-UPF1_hel_ and UPF1 unwind the nucleic acid substrate to the same extent (as determined from the 30-min time point of the reaction), the overall unwinding activity of MOV10_NTD_-UPF1_hel_ is approximately 3-fold higher than that of UPF1 (Fig. 2*A*, compare red and yellow traces). The initial unwinding activity of MOV10_NTD_-UPF1_hel_ is significantly (7-fold) higher than that of UPF1 but slows down after the first 5 min, while UPF1 shows steady unwinding over the entire course of the reaction (Figs. 2*A* and S3*D*). A similar trend is observed upon measurement of RNA-dependent ATP hydrolysis by the two proteins using a coupled enzymatic assay. The RNA-dependent ATPase activity of MOV10_NTD_-UPF1_hel_ is 1.8-fold higher than that of UPF1 (Fig. 2*B*). However, while the unwinding and ATPase activities of MOV10_NTD_-UPF1_hel_ are higher than that of UPF1, they are significantly lower than the activities of the constitutively active UPF1_hel_ protein (Fig. 2, *A* and *B*). These results suggest that the MOV10 NTD also exerts an inhibitory effect on the UPF1 helicase domain. Nevertheless, this effect is weaker than that mediated by the UPF1 CH domain. This difference is likely due to different intramolecular interactions of the UPF1 CH domain or the MOV10 NTD with the helicase core of UPF1.

### Intermolecular interactions of the MOV10-NTD with NMD factors differ from those mediated by the CH domain of UPF1

The core NMD factor, UPF2 engages the UPF1 CH domain in a bipartite interaction, inducing a conformational change that switches the helicase from an inactive RNA-clamping mode to an active RNA-unwinding mode, leading to a stimulation of ATPase and nucleic acid unwinding activities (Fig. 2, *A* and *B*). Apart from enhancing UPF1 activity, UPF2 also promotes phosphorylation of UPF1 by the PI3K-like kinase, SMG1 (27, 28, 29). Given its role in modulating UPF1 in NMD, we proceeded to investigate if UPF2 has a similar impact on MOV10. As a first step, we tested the interaction of MOV10_NTD_-UPF1_hel_ with UPF1 and UPF2. Full-length MOV10 was shown to interact with UPF1 in HEK293 cells. The interaction was weakened, but not abolished, upon treatment of the cell lysate with RNases, pointing to a role of RNA in bridging the interaction (14). We performed an *in vitro* GST pull-down experiment using GST-MOV10_NTD_-UPF1_hel_ as a bait (and GST as a negative control). UPF1 and a near full-length variant of UPF2 (spanning residues 121–1227) were used as preys in this setup. MOV10_NTD_-UPF1_hel_ showed clear binding to UPF2, but not to UPF1, *in vitro* (Fig. 2*C*).

Previous studies mapped the UPF1-binding site of UPF2 to a short stretch of approximately 100 residues (1105–1198), C terminal to the three structured MIF4G (middle of eIF4G) domains (20). To identify the region of UPF2 that binds the MOV10 NTD, we carried out GST pull-downs using GST-MOV10_NTD_-UPF1_hel_ as a bait and a series of UPF2 variants, encompassing or lacking distinct domains as preys (Fig. 2*D*). To ensure that binding of MOV10_NTD_-UPF1_hel_ to UPF2 is attributed to the NTD of MOV10 and not the helicase core of UPF1, we included GST-UPF1_hel_ as a negative control bait. GST-UPF1 served as a positive control in this setup (Figs. 2*E* and S3*E*). As expected, the near full-length UPF2 showed robust binding to GST-UPF1 and GST-MOV10_NTD_-UPF1_hel_ but not to GST-UPF1_hel_. A UPF2 variant encompassing the MIF4G1 and 2 domains did not show any binding to GST-MOV10_NTD_-UPF1_hel_. Variants of UPF2 comprising the MIF4G3 domain (UPF2s and MIF4G3) showed strong binding to MOV10_NTD_-UPF1_hel_ (Fig. 2*E*, lanes 3 and 4). The UPF2s protein which contains the UPF1-binding domain (U1BD) showed weak binding to GST-UPF1_hel_ but stronger binding to GST-UPF1 (Fig. 2*E*, lanes 8 and 12). This is in accordance with the U1BD of UPF2 binding to the CH domain of UPF1. In contrast, the UPF2-MIF4G3 protein shows very weak interactions with both GST-UPF1 and GST-UPF1_hel_ but a strong interaction with GST-MOV10_NTD_-UPF1_hel_ (Fig. 2*E*, compare lanes 4, 9 and 13, and Fig. S3*E*). Taken together, our *in vitro* interaction studies show a direct and specific interaction of the NTD of MOV10 with UPF2, mediated by its MIF4G3 domain.

**Figure 3:**
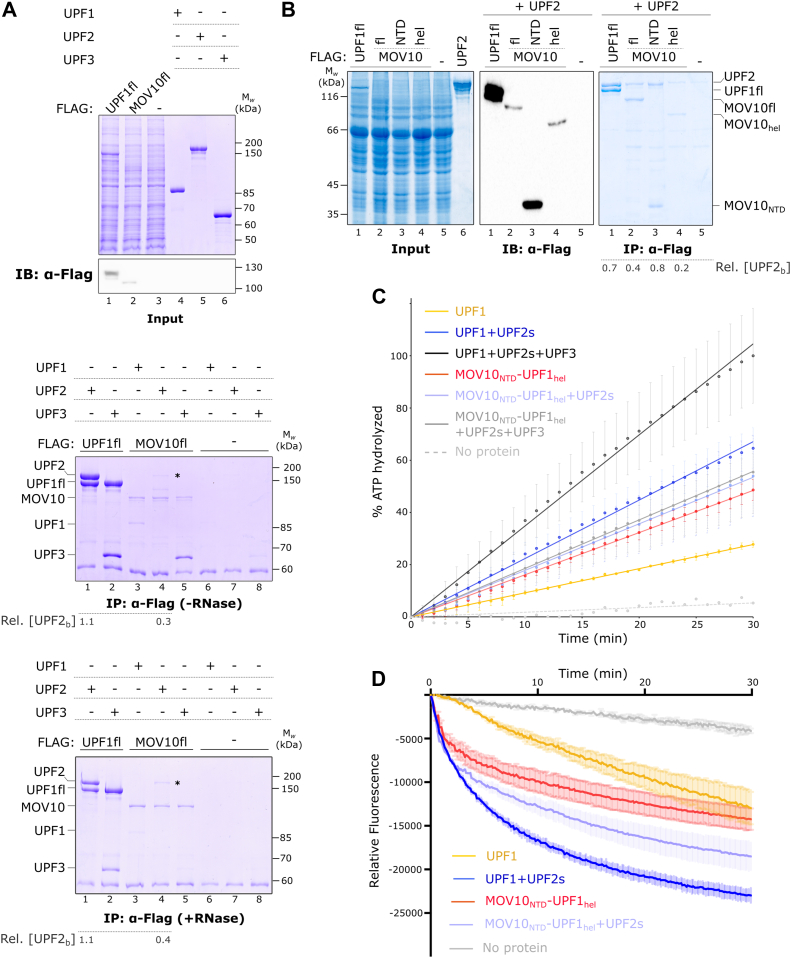
**MOV10 interactors do not influence the catalytic activity of MOV10_NTD_-UPF1_hel_**. *A*, coprecipitation assays of full-length MOV10 and UPF1 with core NMD factors. Flag-tagged full-length UPF1 and MOV10 proteins expressed in HEK293 cells were used as baits, while recombinantly purified UPF1, UPF2, and UPF3 were used as preys (*top panel*). Immunoblotting of HEK293 lysates using an anti-Flag antibody confirmed expression of UPF1 and MOV10. Coprecipitation experiments were performed in the absence and presence of RNase A to capture RNA-dependent (*middle panel*) and RNA-independent interactions (*bottom panel*) of the helicases. UPF1 showed strong binding to both UPF2 and UPF3 in the absence of RNase A, but reduced binding to UPF3 upon RNase A treatment. MOV10 from untreated cell lysates showed strong binding to UPF3, and very weak binding to UPF1 and UPF2. Although the interactions with UPF1 and UPF3 were abolished upon RNase A treatment, binding of UPF2 was slightly enhanced under these conditions, suggesting a direct interaction of MOV10 with UPF2. The identity of the band corresponding to UPF2 (denoted by ∗) was confirmed by tryptic in-gel digestion followed by peptide mass fingerprint analysis using MALDI-TOF-MS (Table S2). Rel. [UPF2_b_] refers to the relative amount of UPF2 protein bound to the helicase bait in each sample in Figure 3, *A* and *B*. *B*, coprecipitation assays of Flag-tagged MOV10 variants with purified UPF2 protein in the presence of RNase A. Flag-tagged full-length UPF1 was used as a positive control. The *left panel* shows the HEK293 lysates of cells expressing UPF1 and MOV10 proteins and the purified UPF2 protein used. Immunoblotting of the cell lysates using an anti-Flag antibody confirmed expression of the UPF1 and MOV10 proteins. The *right panel* shows RNA-independent interactions of UPF2 with UPF1 and MOV10 proteins. Binding of MOV10_NTD_ to UPF2 is comparable to that of MOV10fl, while the binding of MOV10hel is considerably weaker. *C* and *D*, addition of UPF2 does not influence the nucleic-acid unwinding activity or the RNA-dependent ATPase activity of MOV10_NTD_-UPF1_hel_. The experiment, data analysis, and representation were carried out as described in Figure 2. Under similar conditions, a robust enhancement of UPF1 catalytic activity was observed upon addition of UPF2 (and UPF3 in *C*). MOV10, Moloney leukemia virus 10; NMD, nonsense-mediated mRNA decay; UPF1, protein up-frameshift 1.

We sought to validate the interaction of UPF2 with the NTD of MOV10 in context of the full-length MOV10 protein. This was primarily to exclude that the helicase core of MOV10 masks interaction interfaces that prevent binding of UPF2. As full-length MOV10 could not be purified to homogeneity on a large scale, we expressed Flag-tagged human MOV10 (MOV10fl) and as a control, Flag-tagged full-length UPF1 (UPF1fl) in HEK293 cells. The expression of the proteins was verified by immunoblotting with an anti-Flag antibody (Fig. 3*A*, top panel). Small-scale affinity purification using anti-Flag antibody conjugated to agarose resin yielded reasonably pure immobilized proteins that were directly used in co-immunoprecipitation (co-IP) experiments (Fig. 3*A*, middle and bottom panels). The higher expression of Flag-UPF1fl in HEK293 cells resulted in larger amounts of purified protein. Affinity purification of HEK293 lysate containing no Flag-tagged protein (blank) did not yield any significant product (Fig. 3*A*, middle panel, lanes 6–8), corroborating the specificity of this singe-step purification.

To test if full-length MOV10 interacts with core NMD factors, we performed coprecipitation assays using the Flag-tagged proteins immobilized on anti-Flag agarose as baits and purified UPF1, UPF2, and UPF3 proteins as preys. As expected, UPF1fl showed a strong interaction with UPF2. Significant amounts of UPF3 also coprecipitated with UPF1 (Fig. 3*A*, middle panel, lanes 1 and 2). Full-length MOV10 showed a weak interaction with UPF1 and UPF2, and a much stronger interaction with UPF3 (Fig. 3*A*, lanes 3, 4, and 5, respectively). Given the weak intensity of the band corresponding to UPF2 (denoted by ∗ in the middle and bottom panels of Fig. 3*A*), its identity was further verified and confirmed by tryptic in-gel digestion followed by peptide mass fingerprint analysis using MALDI-TOF-MS (Table S2). No bands corresponding to UPF1, UPF2, or UPF3 were detected with the untransfected HEK293 lysate, underscoring the specificity of the interactions observed with UPF1 and MOV10 (Fig. 3*A*, lanes 6–8).

The observed interaction of full-length MOV10 with UPF3 prompted us to test the interaction of the two proteins *in vitro*. UPF3 did not show any appreciable binding to MOV10_NTD_-UPF1_hel_ on its own but was coprecipitated by GST-MOV10_NTD_-UPF1_hel_ in the presence of UPF2 (Fig. S3*F*, lanes 1 and 2). These observations suggest that either UPF3 interacts specifically with the helicase core of MOV10 or that the interaction observed with full-length MOV10 is dependent on RNA. To distinguish between these two possibilities, we repeated the interaction studies with HEK293 cell lysates treated with RNase A (Fig. 3*A*, bottom panel). Under these conditions, the interaction of UPF1 and UPF3 with full-length MOV10 were significantly reduced, while that of UPF2 with full-length MOV10 was marginally enhanced (Fig. 3*A*, compare lanes 3–5 of bottom and middle panels). We next tested the interaction of UPF2 with the NTD and the helicase core of MOV10. To this end, we individually expressed these MOV10 domains in HEK293 cells and performed one-step affinity purification with anti-Flag antibody conjugated resin, as described above (Fig. 3*B*). The overall expression of the MOV10 variants is lower than that of UPF1. Expression of MOV10_hel_ is comparable to that of MOV10fl and much lower than that of MOV10_NTD_ (Fig. 3*B*, lanes 2 and 4 of middle panel). UPF2 coprecipitated with Flag-MOV10fl and Flag-MOV10_NTD,_ and to a much lesser extent (∼4-fold less) with Flag-MOV10_hel_ (Fig. 3*B*, lanes 2–4, right panel). The binding of UPF2 to MOV10_NTD_ is comparable to that of MOV10fl, indicating that the MOV10-UPF2 interaction is driven primarily by the MOV10 N-terminus. These results are consistent with our *in vitro* biochemical studies that suggest a direct interaction between MOV10 and UPF2, mediated by the MOV10 NTD.

The robust activation of UPF1 upon binding UPF2 led us to ask if the interaction of UPF2 with the MOV10 NTD elicits a similar effect on its catalytic activity. Addition of UPF2, alone or together with UPF3, led to no significant change in the RNA-dependent ATPase activity of MOV10_NTD_-UPF1_hel_ (Fig. 3*C*, compare red, light blue, and gray traces, 1.1-fold increase in both cases). This contrasts with UPF1, which shows a robust 2.4-fold increase in ATPase activity upon addition of UPF2 and a further 1.6-fold increase upon addition of both UPF2 and UPF3 (Fig. 3*C*, compare yellow, dark blue, and black traces). A similar trend was observed when assessing the nucleic acid unwinding activities of MOV10_NTD_-UPF1_hel_ and UPF1 in the absence and presence of UPF2; addition of UPF2 led to a marginal increase (1.25-fold) of unwinding activity of MOV10_NTD_-UPF1_hel_ (Fig. 3*D*, compare red and light blue traces). It appears that binding of UPF2 to the NTD of MOV10 does not directly impact the catalytic activity of the helicase core, further highlighting the differences between the UPF1 CH domain and the MOV10 NTD. However, this experimental setup does not allow us to exclude that binding of UPF2 to the MOV10 NTD could still influence the catalytic activity of the MOV10 helicase core.

### Differential intermolecular interactions and regulation correlate with differences in subcellular localization of MOV10 and UPF1

Our observations of the molecular mechanisms, interactions, and regulation of MOV10, and its differences to UPF1 led us to hypothesize that the two helicases might display important functional differences in cells, even in the context of NMD. To test this notion, we studied how these proteins localize to known RNA-rich cytoplasmic bodies, such as processing bodies (P-bodies, PBs) and stress granules (SGs) (30). P-bodies are constitutive membraneless organelles that are rich in RNA decay factors, including NMD proteins, and are thought to be sites of RNA decay, storage, or sequestration to prevent translation (31, 32, 33). Unlike PBs, SG formation is induced upon global translation arrest due to stress conditions (such as heat or oxidative stress) (34, 35). The core of SGs is formed by condensation of mRNPs comprising those mRNAs that are not associated with ribosomes under stress conditions (36).

**Figure 4:**
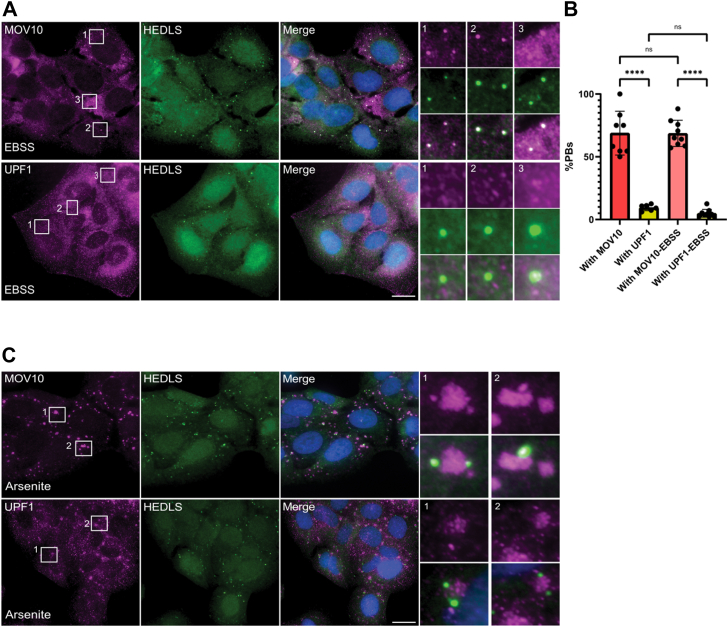
**Localization of MOV10 and UPF1 to cytoplasmic bodies***A*, U2OS cells were treated with EBSS for 8 h, and then stained with anti-Dcp1a antibody to mark PBs (*green*) and with either anti-MOV10 antibody (*top panel*) or anti-UPF1 antibody (*bottom panel*; *magenta*). MOV10 can be seen localizing strongly to PBs while UPF1 does not. DNA Hoechst staining in *blue* marks the nucleus. *B*, quantifications of the percentage of PBs containing MOV10 or UPF1. Values do not change upon cell starvation (EBSS treatment). Data were analyzed using one-way ANOVA with Tukey's post hoc analysis (∗∗∗∗*P* < 0.0001; n.s.= non significant). Data shown as mean±s.d., n = 3 biological replicates, *dots* represent technical replicates. *C*, U2OS cells were treated with arsenite for 1 h, and then cells were stained with anti-Dcp1a antibody to mark PBs (*green*) and either anti-MOV10 antibody (*top panel*) or anti-UPF1 antibody (*bottom panel*, *magenta*). Both proteins localize to stress granules, while only MOV10 can localize to SGs and PBs simultaneously (see Fig. S4). The scale bars represent 10 μm. MOV10, Moloney leukemia virus 10; PBs, processing bodies; SG, stress granule; UPF1, protein up-frameshift 1.

Previous studies that analyzed the PB-proteome suggested that UPF1 and MOV10 are both components of PBs (32). To increase the number of PBs, U2OS cells were grown in medium lacking amino acids (Earle's Balanced Salt Solution, EBSS) (37). The protein HEDLS (also known as EDC4) was used as a PB marker in Figure 4, *A* and *C*. Using fluorescence microscopy, we observed MOV10 in 70% of PBs, regardless of their size, whereas UPF1 was recruited only to very large PBs (5% of PBs) (Fig. 4*A*). Although amino acid starvation conditions increased PB numbers (from ∼1–2 per cell to ∼5–8), it did not change the percentage of PBs containing MOV10 and UPF1 (Fig. 4*B*). As the amount of UPF1 in HEK293 cells is almost 8-fold that of MOV10 (38), it can be assumed that not all NMD targets of UPF1 are bound by MOV10. Accordingly, Gregersen et al. found that MOV10 binds only a subset of UPF1 NMD targets (14). Not surprisingly therefore, MOV10 and UPF1 do not share a significant overlap in their subcellular localization.

We next proceeded to analyze the localization of the helicases relative to SGs that form upon oxidative stress, induced by treating cells with sodium arsenite. Both MOV10 and UPF1 were found to be recruited to all SGs (Figs. 4*C* and S4). Interestingly, analysis of SG formation showed that MOV10 can be found in both PBs and SGs simultaneously and can be seen docking onto SGs early after arsenite treatment, while it resides in the PB (Fig. 4*C*, insets of top panel). This suggests that MOV10 undergoes a rearrangement in protein-protein/RNA interactions to relocalize from PBs to SGs.

**Figure 5:**
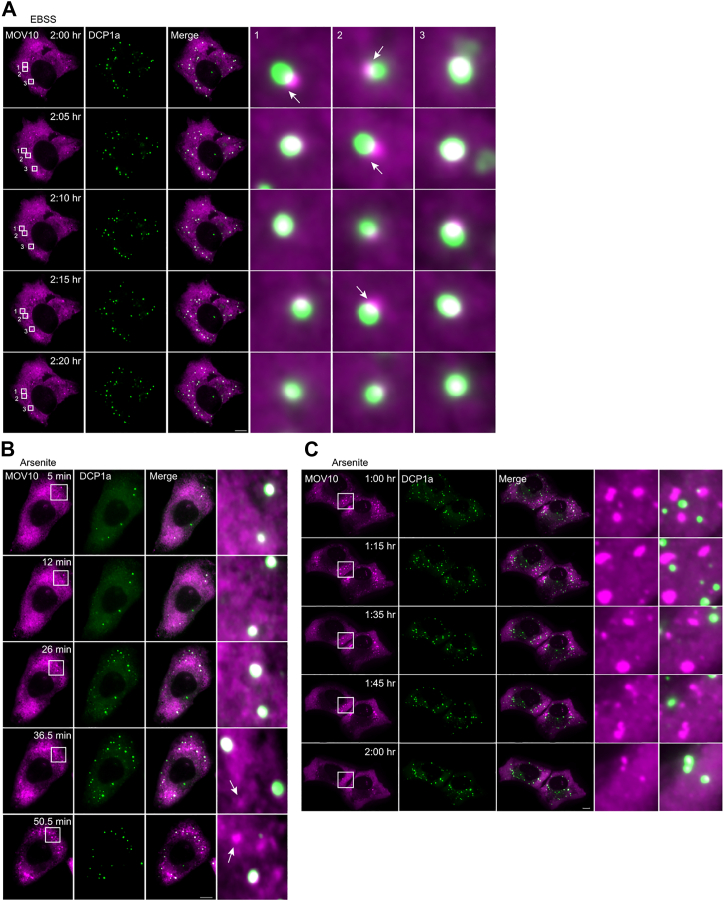
**Live**-**cell imaging of MOV10 with cytoplasmic bodies reveals dynamic movement during stress**. *A*, frames from time-lapse movies showing PBs in U2OS cells expressing MOV10-RFP and DCP1a-GFP after 2 h EBSS starvation. MOV10 can be seen embedded within the PB, while occasionally moving out to the periphery. *B*, frames from time-lapse movies of U2OS cells expressing MOV10-RFP and DCP1a-GFP under treatment with arsenite (0.5 mM). As SGs begin to form, MOV10 can be seen aggregating at the newly formed granules. *C*, frames from time-lapse movies of U2OS cells expressing MOV10-RFP and DCP1a-GFP after treatment with arsenite (0.5 mM). As SGs begin to dissolve after ∼1 h 45 min, MOV10 returns to the PBs from the SGs. The scale bars represent 10 μm. MOV10, Moloney leukemia virus 10; PBs, processing bodies; SG, stress granule.

To investigate the localization patterns in further depth, we employed live-cell imaging to track the dynamics of these proteins. Amino acid starvation (up to 3.5 h) resulted in significant recruitment of MOV10 to PBs (Fig. 5*A*). The decapping factor GFP-DCP1a was used as a PB marker. RFP-MOV10 was observed to be mostly embedded in the PBs; however, in some frames the protein was seen at the periphery of the PB (marked by white arrows), possibly as it entered the phase-separated body. To highlight this, three PBs are shown in the zoomed images, one where MOV10 was initially disassociated from the PB and reentered it at a later time point (Fig. 5*A*, inset 1), one where we observed a more frequent dissociation and reassociation of MOV10 with the PB (inset 2), and one where MOV10 was firmly embedded in the PB (inset 3). Although MOV10 initially localized in PBs, it relocated to SGs at around 40 min after arsenite stress induction (Fig. 5*B*). This is evident from the fact that not all granules containing MOV10 colocalized with the DCP1a-stained granules that denote PBs (Fig. 5*B*, see arrows in the two lower panels). It is known that after a few hours of arsenite exposure, SGs dissolve to release their contents back into the bulk cytosol. We chose, therefore, to also image cells after arsenite exposure until dissolution (2 h post treatment). We found that MOV10 colocalized with DCP1a at the 2 h time point, indicating that its return to PBs occurred once the SGs had dissolved (Fig. 5*C*, bottom panel). It appears therefore that MOV10 cycles between PBs and SGs depending on the physiological condition, relocating from PBs to newly formed SGs upon induction of oxidative stress and back to PBs upon SG dissolution, after cessation of oxidative stress. In contrast, UPF1 remained diffuse throughout the cytoplasm during amino acid starvation but localized to SGs upon their formation after arsenite exposure (Fig. S5, *A* and *B*).

**Figure 6:**
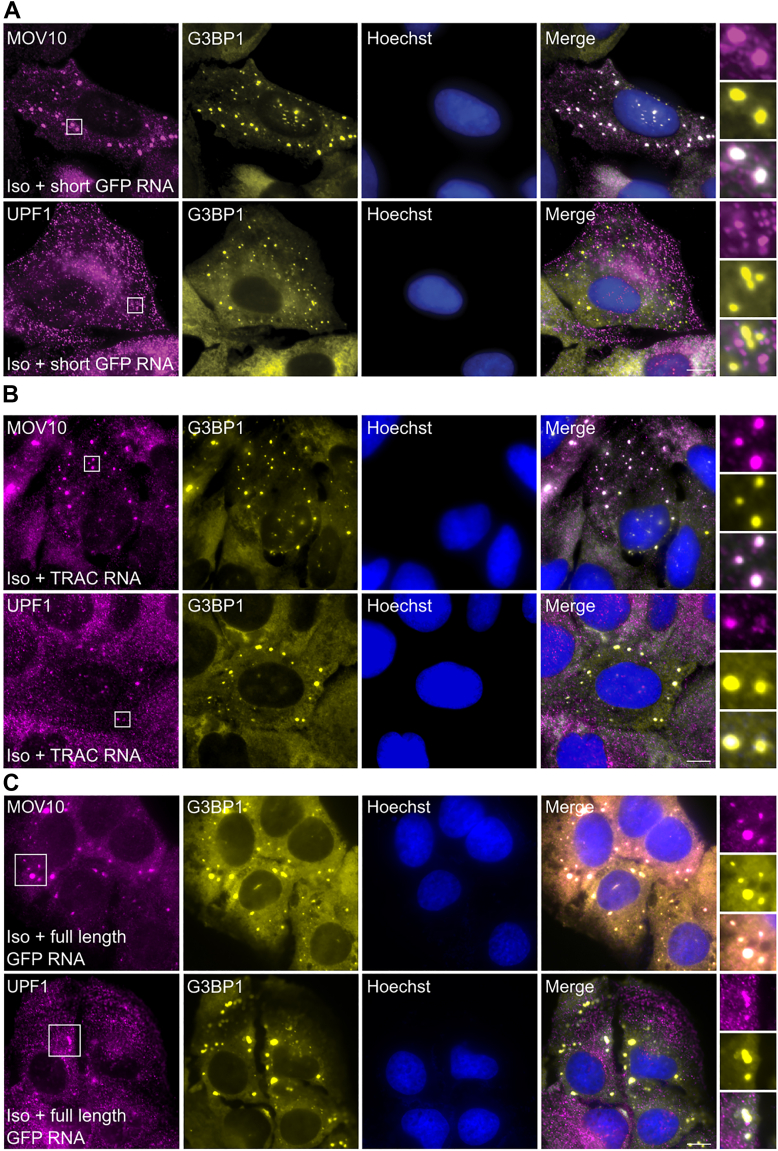
**Delivery of synthetic mRNA induces SG formation revealing differential interactions of UPF1 and MOV10.***A*, U2OS cells were treated with isoginkgetin (Iso, 50 μM) for 5 h, transfected with *in vitro*-transcribed mRNA (1 μg) encoding a short GFP (short GFP RNA) and fixed after 1.5 h. Cells were stained using anti-G3BP1 antibody (*yellow*), and either anti-MOV10 antibody (*top panel*) or anti-UPF1 antibody (*bottom panel*; *magenta*). Although MOV10 localized to the SGs, UPF1 did not. *B*, U2OS cells were treated with isoginkgetin (Iso, 50 μM) for 5 h, transfected with *in vitro*-transcribed mRNA (1 μg) encoding the TCR alpha chain (TRAC) and fixed after 1.5 h. Cells were stained as described above for (*A*). Both MOV10 and UPF1 localized to SGs. DNA Hoechst staining is shown in *blue*. The scale bars represent 10 μm. *C*, U2OS cells were treated with isoginkgetin (Iso, 50 μM) for 5 h, transfected with *in vitro*-transcribed mRNA (1 μg) encoding full-length GFP (full-length GFP RNA) and fixed after 1.5 h. Cells were stained as described above for (*A*). Both MOV10 and UPF1 localized to SGs. DNA Hoechst staining is shown in *blue*. The scale bars represent 10 μm. MOV10, Moloney leukemia virus 10; SG, stress granule; UPF1, protein up-frameshift 1.

Cytosolic RNA depletion, achieved by treating cells with splicing inhibitors, followed by transfection of synthetic RNA can induce the formation of SGs without oxidative stress (39). To determine how the lack of RNA partners would alter the localization of the helicases, we depleted cytosolic RNAs by treating cells with the splicing inhibitor isoginkgetin (39) and transfected an RNA that encodes a shortened GFP transcript (250 bp, short GFP RNA), an RNA that encodes the α subunit of the T-cell receptor, TRAC (800 bp, TRAC RNA), and an RNA that encodes full-length GFP (922 bp, full-length GFP RNA). Staining of cells using an oligo-dT probe showed a strong decrease in signal in the cytoplasm upon treatment with isoginkgetin, confirming the reduction of poly(A) mRNA in the cytoplasm (Fig. S6*A*). After introduction of short GFP-RNA into the isoginkgetin-treated cells, MOV10 continued to be recruited to the newly forming granules and was found associated with all SGs (Fig. 6*A*, top panels). The protein G3BP1 was used as a SG marker in these experiments. Cells that did not take up the synthetic RNA showed no SGs but still contained PBs to which MOV10 was localized (Fig. S6*B*), indicating that recruitment of MOV10 to PBs is mostly driven by protein-protein interactions.

In contrast to MOV10, after transfection of the short GFP-RNA, UPF1 was largely absent from SGs (Fig. 6*A*, bottom panels). Only a few large granules (∼3–4 micron) contained UPF1. This effect is independent of the splicing inhibitor used to deplete endogenous cytoplasmic mRNA, as we observed the same behavior in cells treated with pladienolide B (PLB) and transfected with short GFP-RNA (Fig. S7*A*). To confirm that the transfection of synthetic short GFP RNA does not deter UPF1 from entering granules, we transfected untreated cells with this RNA. Indeed, both UPF1 and MOV10 were found in all granules of all sizes (Fig. S7*B*). Unlike with short GFP-RNA, UPF1 was found in the SGs of isoginkgetin-treated cells that were transfected with TRAC-RNA and full-length GFP RNA (Fig. 6, *B* and *C*, bottom panels). The dependency of UPF1 localization on the exogenous RNA introduced in cells suggests that recruitment of UPF1 to granules is primarily driven by its interactions with RNA.

The algorithm catRAPID 2.1, which examines the possible interactions between specific proteins and RNA, predicted an interaction of UPF1 and MOV10 with the TRAC transcript (a high score of 69.89 and 66.02, respectively) but not with the short GFP-RNA. A transcript encoding for full-length GFP (full-length GFP RNA) was also predicted to bind both UPF1 and MOV10 (Fig. S7, *C* and *D*). However, MOV10 localized to SGs, independent of the nature of the exogenous RNA transfected. These results suggest that while RNA-protein interactions dictate UPF1 localization to SGs in cells, the recruitment of MOV10 to RNA-rich granules is not dependent on its interaction with RNA.

**Figure 7:**
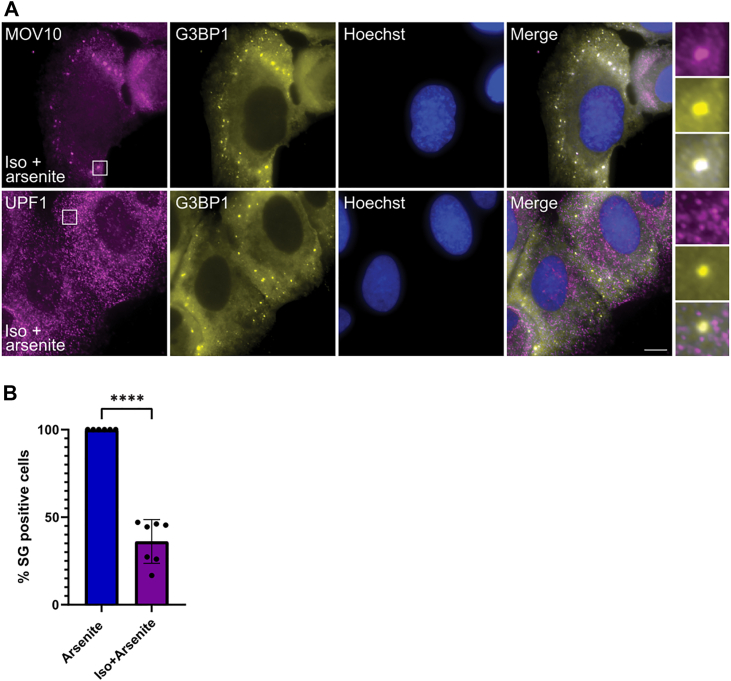
**Unraveling the role of mRNA in recruitment of MOV10 and UPF1 to SGs.***A*, U2OS cells were treated with isoginkgetin (Iso, 50 μM) for 5 h overall, transfected with short GFP RNA (0.75–1 μg) for 1.5 h and treated with arsenite (0.5 mM) for 1 h before fixation. Cells were stained for G3BP1 and MOV10 or UPF1 as described above. Regardless of SG induction, UPF1 did not localize to SGs after RNA depletion. DNA Hoechst staining is shown in *blue*. The scale bars represent 10 μm. *B*, quantification of SG positive cells after arsenite exposure and with pretreatment with isoginkgetin. Data were analyzed using a two-tailed paired *t*-test (∗∗∗∗*P* < 0.0001). Data shown as mean±s.d., n = 3 biological replicates, *dots* represent technical replicates. MOV10, Moloney leukemia virus 10; SG, stress granule; UPF1, protein up-frameshift 1.

We next induced oxidative stress in cells where the cytoplasmic RNA was depleted by treatment with isoginkgetin (Fig. 7*A*). Due to lack of RNA (native or synthetic), only about 35% of cells succeeded in forming SGs (Fig. 7*B*). Analysis of the cells containing SGs revealed MOV10 present in all granules, while UPF1 was absent from them (Fig. 7*A*). This observation is consistent with our suggestion that recruitment of UPF1 to SGs is dependent on its interactions with RNA, while localization of MOV10 is likely driven by protein-protein interactions that are distinct from those mediated by UPF1.

**Figure 8:**
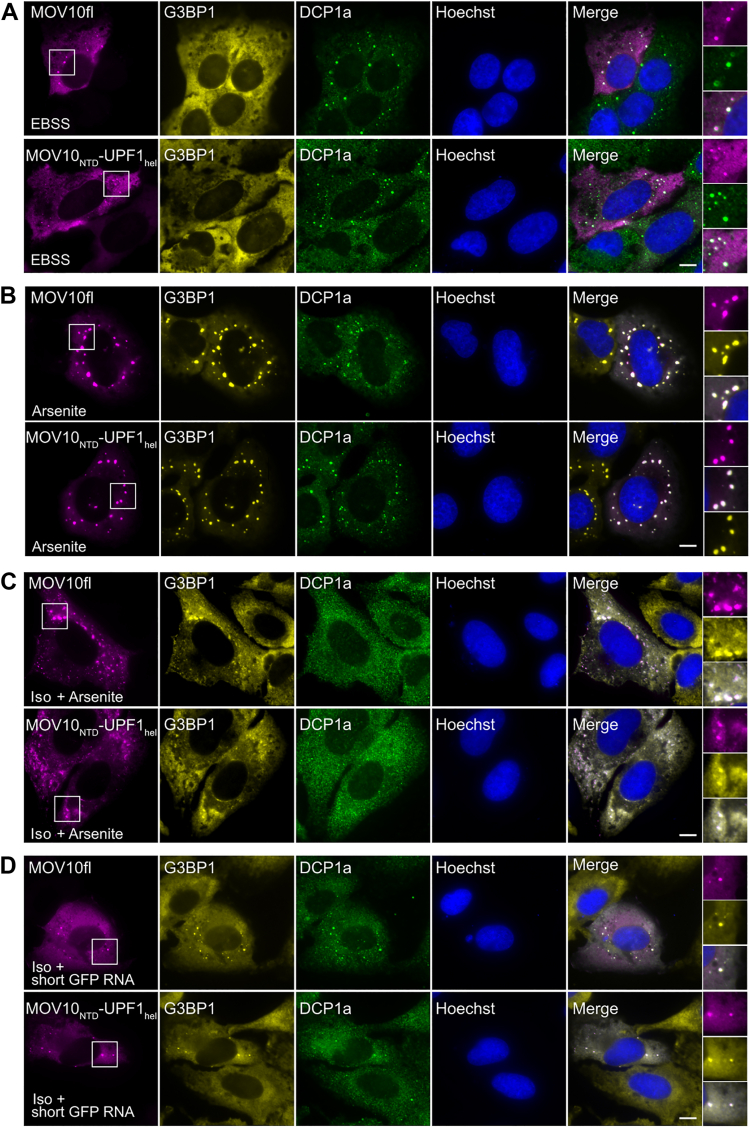
**The MOV10 N-terminal domain is determinative of its subcellular localization.***A*, U2OS cells were transfected with either MOV10fl (*top*) or MOV10_NTD_-UPF1_hel_ (*bottom*) and then treated with EBSS for 8 h. After fixation, cells were stained with anti-G3BP1 to mark SGs (*yellow*) and anti-Hedls to mark PBs (*green*). Both constructs succeeded in localizing to PBs. The scale bars represent 10 μm. *B*, cells were transfected and stained as in *A*, but treated with arsenite (0.5 mM) to induce SG formation. Both constructs succeeded in localizing to SGs. *C*, cells were transfected and stained as in (*A*), but were treated with isoginkgetin (Iso, 50 μM) for 5 h overall, and treated with arsenite (0.5 mM) 1 h before fixation. In SG positive cells, both constructs succeeded in localizing to the SGs. *D*, cells transfected and stained as in (*A*) but were treated with isoginkgetin (Iso, 50 μM) for 5 h overall, and transfected with transfected with *in vitro*-transcribed short GFP RNA (1 μg) and fixed after 1.5 h. In SG positive cells, both constructs succeeded in localizing to the SGs. MOV10, Moloney leukemia virus 10; PBs, processing bodies; SG, stress granule; UPF1, protein up-frameshift 1.

Our biochemical data point to the divergent N-terminal domains as being responsible for the differential interactions mediated by the two helicases. This led to the question if localization of the chimeric protein MOV10_NTD_-UPF1_hel_ is dictated by protein-protein interactions mediated by the MOV10 NTD or protein-RNA interactions mediated by the UPF1 helicase core. To address this, we analyzed the localization of MOV10_NTD_-UPF1_hel_-GFP in U2OS cells under different cellular treatment conditions. DCP1a and G3BP1 served as markers for PBs and SGs, respectively, in these experiments. Like MOV10fl, MOV10_NTD_-UPF1_hel_ is present in PBs formed upon EBSS treatment and localizes to SGs upon induction of oxidative stress (Fig. 8, *A* and *B*). Unlike UPF1, depletion of cytoplasmic RNA by isoginkgetin treatment, followed by arsenite treatment or transfection of a short GFP RNA did not deter localization of MOV10_NTD_-UPF1_hel_ to SGs. Our results show that the behavior of the MOV10_NTD_-UPF1_hel_ is very similar to that of MOV10fl, indicating that the localization of this protein in cells is dictated by protein-protein interactions mediated by the MOV10 N-terminal domain. The impact of these interactions on localization to RNA-rich condensates raises the question if RNA binding and catalytic activity of MOV10 influence its localization at all.

To address this, we transfected a catalytic mutant of MOV10 (MOV10 K530A) that binds RNA but not ATP, and therefore shows no ATP hydrolysis or RNA unwinding activity (14). We find that this MOV10 mutant localizes to PBs upon EBSS treatment but does not move to SGs upon induction of oxidative stress in the presence or absence of cytoplasmic mRNA (Fig. S8). Since the interactions mediated by MOV10 proteins should be unchanged upon mutation of the ATP-binding site, it follows that perturbation of the cycle of RNA-dependent ATP hydrolysis by MOV10 affects its ability to shuttle from PBs to SGs. Studies on UPF1 show that ATP hydrolysis stimulates dissociation of the helicase from RNA (40). We speculate that MOV10 functions in a similar manner to release its RNA substrate upon ATP hydrolysis. Accordingly, a MOV10 variant that is incapable of hydrolysing ATP remains stably bound to RNA, preventing its shuttling between two RNA-rich condensates. Taken together, the above results suggest that a complex and distinct set of protein-protein/RNA interactions drive the localization of MOV10 and UPF1 to cytoplasmic bodies in cells.

## Discussion

Although the RecA domains of RNA helicases are highly conserved and encompass the motifs necessary for RNA and ATP binding, the divergent regulatory domains flanking the RecA domains expand the functionality of each helicase and provide specificity in a cellular context. The regulatory domains of the SF1 RNA helicases UPF1, SETX, and IGHMBP2 are diverse in length, sequence, and structure and play important roles in modulating their catalytic activity as well as their functions in cells. In contrast, very little was known about the regulatory domain of the SF1 RNA helicase, MOV10. In this study, we investigated the role of the NTD of MOV10 in comparison to the CH domain of UPF1 to explore the functional differences between MOV10 and UPF1. We found similarities as well as differences in the intramolecular and intermolecular interactions mediated by the MOV10 NTD and UPF1 CH domain, which we speculate could dictate the differences in their physiological roles in cells.

### Impact of the MOV10-NTD on the helicase core

The chimeric MOV10_NTD_-UPF1_hel_ protein was used *in lieu* of the isolated MOV10-NTD to study the interactions mediated by this domain, independent of the MOV10 helicase core. This strategy has been previously employed to study insoluble or proteolytically unstable proteins, where creation of a chimeric variant remarkably improved the biochemical behavior of the protein (41). Comparison of Alphafold 3-predicted structures of MOV10fl and MOV10_NTD_-UPF1_hel_ show a very similar fold of the MOV10 NTD, although the orientation of the NTD with respect to the helicase core differs in both structures. The RNA-binding affinity of the chimeric MOV10_NTD_-UPF1_hel_ helicase in the absence of nucleotide was very similar to that of UPF1 and UPF1_hel_, indicating that fusion of the MOV10 NTD to the UPF1 helicase core did not alter the RNA-binding affinity of the chimeric helicase. We, as well as other researchers, previously observed a decrease in RNA-binding affinity of the UPF1 helicase in the presence of ATP (18, 19, 22, 26). UPF1 variants containing or lacking the CH domain showed a similar trend. This was attributed to the ATP-dependent repositioning of a regulatory loop within domain 1B, which swings into the RNA-binding pocket when the helicase binds ATP and swings out upon ATP hydrolysis, allowing UPF1 to translocate on RNA (26). In contrast to UPF1, addition of a nonhydrolyzable ATP analog did not result in a change in RNA-binding affinity of MOV10_NTD_-UPF1_hel_. We speculate that the MOV10 NTD either prevents the regulatory loop from swinging into the RNA-binding pocket or stabilizes it in an “open” position away from the RNA-binding pocket, thereby minimizing any obstruction in RNA binding. The effect of the MOV10 NTD on RNA-binding by the helicase core raises the question of how the catalytic activity of the chimeric helicase is regulated. The basal ATPase and unwinding activities of UPF1 are quite low due to the autoinhibition by its CH domain. Autoinhibition results from an intramolecular hydrophobic interaction between the CH and RecA2 domains that maintains UPF1 in a “closed” RNA-clamping conformation. Recent cryoEM structures of full-length UPF1 in RNA-bound and unbound states reveal that UPF1 is in an “open” conformation (CH domain positioned away from RecA2) in the absence of RNA, and switches to the closed conformation only upon binding RNA (42). The residue of the UPF1 RecA2 domain that supports the closed conformation of UPF1 (I757) is not conserved in MOV10. Moreover, the fold of the MOV10 NTD (as derived from the Alphafold 3 predictions) is distinct from the UPF1 CH domain. We speculate that the interactions mediated by the MOV10 NTD with the UPF1 helicase core are very different from that of the CH-RecA2 interactions in UPF1. It appears that the MOV10 NTD cannot engage the UPF1 helicase core to inhibit it effectively, resulting in an enzyme whose constitutive activity is higher than that of UPF1. However, the RNA-dependent ATPase activity of MOV10_NTD_-UPF1_hel_ protein is not as high as UPF1_hel_, suggesting that the MOV10-NTD might partially inhibit the UPF1 helicase core *via* other mechanisms, possibly arising from unusual interactions mediated by the MOV10 NTD with the UPF1 helicase core. Full-length MOV10 expressed and purified from mammalian cells was also shown to unwind RNA duplexes, but its activity was not compared to the basal or stimulated unwinding activity of UPF1 in this study.

### Intermolecular interactions mediated by MOV10 NTD

Autoinhibition of UPF1 by its CH domain is relieved upon binding to UPF2. UPF2 uses its C terminal natively unstructured U1BD to engage UPF1. Upon binding UPF1, the U1BD folds into an alpha-helix and a beta-hairpin that are connected by a flexible linker. Although this region of UPF2 is necessary and sufficient for binding, robust activation of UPF1 is achieved only in the presence of the structured MIF4G3 domain that precedes the U1BD (43). The helicase core of UPF1 does not show strong interactions with UPF2. Our biochemical analysis of the interaction of MOV10_NTD_-UPF1_hel_ with UPF1 and UPF2 showed a specific binding of MOV10_NTD_-UPF1_hel_ to UPF2. The interaction was mapped to the UPF2-MIF4G3 domain and is mediated by the MOV10 NTD, as UPF1 and UPF1_hel_ show very little binding to the UPF2-MIF4G3 domain. Binding of UPF2 to MOV10_NTD_-UPF1_hel_ does not alter the catalytic activity of the chimeric helicase, as observed for UPF1. It appears that the role of the MOV10_NTD_-UPF1_hel_:UPF2 interaction is distinct from that of the UPF1:UPF2 interaction, where binding to UPF2 leads to an enhancement in catalytic activity of UPF1. UPF2 is also thought to mediate phosphorylation of UPF1 in context of a larger dynamic complex encompassing the core NMD factor UPF3 and the EJC (28). The presence of an EJC downstream of a termination codon is a signal for NMD that integrates the roles of the stalled ribosome and associated factors that mediate UPF1 phosphorylation, the core NMD factors UPF1-2-3 and downstream NMD factors such as the endoribonuclease SMG6 (21, 44, 45, 46, 47). Our observations of the interaction of MOV10_NTD_-UPF1_hel_ with UPF2 led us to isolate full-length MOV10 from mammalian cells and test its interaction with known NMD factors. Full-length MOV10 showed a weak interaction with UPF2 and a stronger interaction with UPF1. Surprisingly, a very strong interaction with UPF3 was observed. The interaction of MOV10 with UPF1 and UPF3 are strongly dependent on RNA as RNase treatment of the cell lysate led to a loss of binding. In contrast, a stronger binding to UPF2 was observed upon RNase treatment, particularly with the MOV10_NTD_ protein expressed in HEK293 cells, corroborating the interaction between MOV10_NTD_-UPF1_hel_ and UPF2 observed *in vitro*. Although no direct interaction was observed between MOV10_NTD_-UPF1_hel_ and UPF3 *in vitro*, UPF3 coprecipitated with MOV10_NTD_-UPF1_hel_ in the presence of UPF2. UPF2 acts as an adaptor between MOV10 and UPF3, presumably using different surfaces of its MIF4G3 domain to simultaneously bind UPF3 and MOV10. The RNA-dependent interaction of MOV10 with UPF3 raises the question if this helicase can bind the EJC. Immunoprecipitation mass spectrometry experiments to determine RNA-dependent interactors of MOV10 identified the DEAD-box protein EIF4A3, a core component of the EJC, as a binding partner (14). It is possible that MOV10 interacts with EJC-bound UPF3 *via* UPF2 and is tethered to an NMD substrate *via* this mechanism. Although the intramolecular and intermolecular interactions mediated by the MOV10-NTD are distinct from UPF1, they appear to cluster on a subset of NMD-related factors that also bind UPF1.

### Distinct interactions mediated by MOV10 and UPF1 dictate their subcellular localization

UPF1 has a predominantly decay-centric role in mammalian cells and is involved in several pathways apart from NMD, where it is thought to remodel the target mRNP and facilitate mRNA degradation (48). MOV10 appears to have a broader impact on RNA processing as it is also involved in inhibition of viral replication and microRNA-mediated regulation of gene expression (13, 49, 50). To obtain a global view of the functional differences between MOV10 and UPF1, we investigated the subcellular localization of the two helicases using fluorescence microscopy. In particular, we focused on their accumulation in two distinct membraneless organelles, P-bodies and SGs, in different conditions. A high throughput analysis of the PB-proteome previously identified both MOV10 and UPF1 as components of PBs, although the relative abundance of MOV10 in PBs was ∼4.5-fold higher than that of UPF1 (32). Our studies show that MOV10 is a core component of PBs and localizes to SGs only at a later time point of stress induction. This is in line with previous studies that report spatial, compositional, and functional cross talk between PBs and SGs (51). UPF1 is seldom present in PBs but more readily recruited to SGs. Although both PBs and SGs are RNA-rich compartments, we found that localization of MOV10 to PBs and SGs remains unaffected by loss of endogenous cytoplasmic RNA and is possibly driven by protein-protein interactions. The chimeric helicase MOV10_NTD_-UPF1_hel_, like MOV10fl, localizes to PBs and SGs in an RNA-independent manner, furthering supporting the model that protein-protein interactions mediated by the MOV10 NTD dictate its subcellular localization. This contrasts with UPF1 that localizes to SGs only in the presence of RNA that it can bind to, suggesting that its recruitment to SGs is likely dictated by protein-RNA interactions. Accordingly, many RNA-independent MOV10 interactors were found to be enriched in PBs. Nevertheless, the catalytic activity of MOV10 was found to be essential for its release from PBs, indicating that the dynamic exchange of MOV10 between RNA condensates requires ATP hydrolysis. The release of RNA from one RNA-rich condensate and its transfer to another was previously shown to be regulated by the ATP hydrolysis activities of DEAD-box proteins across all organisms (52). We propose that ATP hydrolysis also determines the release of RNA-dependent ATPases (helicases) from RNA-rich condensates to allow their entry into other compartments under different cellular conditions.

Our studies highlight the differences in the MOV10 and UPF1 regulatory domains that lead to differences in regulation of catalytic activity *via* distinct intramolecular and intermolecular interactions. We speculate that these differences influence the physiological functions of the two helicases, in part by influencing their subcellular localization to PBs and SGs under different conditions. PBs are centers of active decay and are also thought to be sites of storage of translationally repressed mRNAs (37, 53). MOV10 plays a role in mRNA decay and translational repression through its association with NMD factors and microRNA-mediated regulatory factors, respectively. Whether MOV10 and UPF1 act collectively to mediated NMD of select targets in PBs remains a topic for further investigation.

## Experimental procedures

### Cloning, protein expression, and purification

The UPF1, UPF2, and UPF3 expression plasmids used in this study were available in the lab. Human full-length MOV10 was subcloned into a baculo expression vector (pFastBac Hta) from a mammalian expression plasmid while the MOV10-UPF1 chimeric constructs were generated by overlapping PCR from the corresponding MOV10 and UPF1 fragments. Primers used for generating the N-terminal MOV10 fragment and the C-terminal UPF1 helicase core fragment corresponding to MOV10_NTD_-UPF1_hel_ are as follows:

5′-ccaggggcccgactcgatgcccagtaagttcagc-3′, 5′-actccctgcagcacctgcagatccgtttccggtgatccg-3′, 5′-gcaggtgctgcagggagtggagctcggtacgaggacgcctac-3′, 5′-gcaaagcaccggcctc gttagctgaactgcatgag-3′.

The first and fourth primers were used for the overlapping PCR.

The human UPF1, UPF2, UPF3, and MOV10_NTD_-UPF1_hel_ chimeric proteins (except those indicated below), were expressed as 6 × His, His-Thioredoxin (Trx) or His-GST fusions in *E. coli* BL21 (*DE3*) STAR pRARE or BL21 (*DE3*) Gold pLysS cells. Cells expressing recombinant proteins were lysed using buffer A (50 mM Tris–HCl pH 7.5, 10% glycerol, 1 mM MgCl_2_, 1 μM ZnCl_2_, 100 mM urea, and 10 mM imidazole) supplemented with 500 mM NaCl, 1 mM PMSF, and DNase I (lysis buffer). The proteins were enriched from the crude lysate by Ni^2+^-affinity chromatography. An additional wash step using buffer A supplemented with 1 M NaCl, 10 mM MgCl_2_, 50 mM KCl, and 2 mM ATP was performed to ensure removal of contaminating chaperones. The one-step purified proteins were subjected to a further purification step using a HiTrap Heparin Sepharose HP column (GE Healthcare). All proteins were finally purified by size-exclusion chromatography (SEC) in SEC buffer (20 mM Tris–HCl/Hepes pH 7.5, 150  mM NaCl, 5% glycerol, 1 mM MgCl_2_, 1 μM ZnCl_2_, and 2 mM DTT). UPF2 CT (residues 1015–1227) was subjected to SEC directly after Ni^2+^-affinity chromatography. Full-length MOV10 was expressed in Sf9 cells cultured in Sf900 media and infected with the MOV10 virus at a 1:1000 v/v ratio, and purified as described above.

### Fluorescence anisotropy

To determine the affinity of UPF1 or MOV10_NTD_-UPF1_hel_ for RNA, 5 nM of a 12-mer poly(U)-RNA (U_12_) labeled with 6-FAM at its 5′-end was mixed with increasing concentrations of protein in FA-binding buffer (20 mM Hepes pH 7.5, 100 mM NaCl, 1 mM MgCl_2_, and 100 μg/ml bovine serum albumin [BSA]) for 30 min at room temperature. For measurements performed in the presence of ATPγS, the nucleotide was added to a final concentration of 1 mM. Briefly, 40 μl of each sample was transferred to a black 384-well plate (PerkinElmer OptiPlate 384-F) and fluorescence polarization was measured with a Tecan Spark plate reader at 25 °C. The reading obtained in the absence of protein was considered as background and subtracted from all FA values. FA was normalized against the value obtained for the highest protein concentration. The data shown are an average of at least three independent experiments and were fitted to an equation representing one site-specific binding with Hill slope in GraphPad Prism 10. Error bars and the error associated with the reported *K*_D_ denote SDand SEM, respectively.

### ATPase assays

The ATPase activity of UPF1, UPF1_hel_ or MOV10_NTD_-UPF1_hel_ was determined by detecting the amount of inorganic phosphate released upon ATP hydrolysis by the proteins using a coupled colorimetric assay system (EnzChek Phosphate Assay Kit). The proteins were preincubated with 2  μg poly(U) RNA of variable length (20–200 nucleotides), 40  nmol MESG (2-amino-6-mercapto-7-methylpurine ribonucleoside), and 0.5  U purine-nucleoside phosphorylase in reaction buffer (50  mM Mes pH 6.5, 50  mM potassium acetate, 5  mM magnesium acetate, and 2  mM DTT) at 30 °C for 30 min. The reaction was initiated by the addition of ATP to a final concentration of 1 mM. Inorganic phosphate released from ATP hydrolysis reacted with MESG to produce 2-amino-6-mercapto-7-methylpurine, which was detected by measuring absorbance at 360  nm on a Tecan Spark plate reader. The reaction was monitored over a 60 min period at 60-s intervals. The amount of UPF1 or MOV10_NTD_-UPF1_hel_ in every experiment was kept constant, and UPF2 or UPF3 was added in 1.5-fold excess, wherever indicated. The end point of the reaction (30-min time point) of the sample with highest ATPase activity (UPF1-UPF2_S_ or UPF1-UPF2_S_-UPF3) was set to 100%, and all other values were normalized to this maximal value.

### Analysis of protein-protein interactions by GST pull-down

Equal amounts of bait and prey proteins (8–12 μg) were mixed and diluted in GST-PD buffer (20 mM Hepes pH 7.5, 100 mM NaCl, 10% Glycerol, and 0.1% NP40) to a total volume of 40 μl. Five percent of each protein mixture was analyzed as input. The reaction mixture was incubated at room temperature for 45 min, following which 15 μl of preequilibrated 50% slurry of Glutathione Sepharose resin (Macharey-Nagel) was added to capture the protein complexes. The mixture was further supplemented with 200  μL of GST pull-down buffer and incubated at 4  °C for 1 to 1.5 hours with mixing. The beads were extensively washed with GST pull-down buffer and incubated with 20 μl elution buffer (50 mM Hepes pH 7.5, 150 mM NaCl, 5% Glycerol, 2 mM DTT, and 30 mM reduced glutathione) for 10 to 15 min at 30 °C. Inputs and eluates were analyzed by SDS-PAGE and Coomassie brilliant blue staining. Quantification of protein levels in SDS gels was done by performing a densitometric analysis of the bands using ImageJ.

### Fluorescence-based nucleic acid-unwinding assay:

The sequences of the RNA and DNA strands of the RNA:DNA hybrid and that of the trap DNA strand are as follows:

RNA strand: 5′-GGGACACAAAACAAAAGACAAAAACACAAAACAAAAGACAAAAACACAA-AACAAAAGACAAAAAGCCAAAUUACCGUGUGCGUACAACUAGCU-3′; Complementary DNA strand: 5′- AGCTAGTTGTACGCACAC -3′; Trap: 5′-GTGTGCGTACAACTAGCT-3′.

The complementary DNA strand of the hybrid was labeled with Alexa Fluor 488 at the 5′-end to generate a fluorescence labeled substrate. The complementary trap DNA strand was labeled with Black Hole quencher 1 at the 3′-end. The RNA strand used to prepare the RNA:DNA hybrid was generated by *in vitro* transcription from a linearized double-stranded (ds) DNA template. The RNA:DNA hybrid was freshly prepared each time by mixing the DNA and RNA strands in a 7:11 ratio with 2 mM magnesium acetate and 1× unwinding buffer (10 mM Mes pH 6.5, 50 mM potassium acetate, and 0.1 mM EDTA) at 95 °C for 3 to 4 min, followed by cooling on ice to anneal the two strands. The reaction mixture contained 75 nM of nucleic acid substrate in 1× unwinding buffer, 2 mM magnesium acetate, 2 mM DTT, and 300 nM of helicase (and 600 nM UPF2 wherever indicated). The reactions were incubated at 25 °C for a total of 20 min. Trap DNA was added to a final concentration of 0.56 μM. The reaction mixtures were transferred to a 384-well plate (PerkinElmer OptiPlate 384-F) where ATP (final concentration of 2 mM) was added to each well using the injector module of the Spark multimode microplate reader (Tecan Life Sciences). The reaction was monitored for 30 min at 30 °C at 10-s intervals. The measured fluorescence intensities were normalized to the 0-time point (baseline) for each condition to obtain relative fluorescence.

### Cell culture and co-IP

To analyze the interactions of UPF1, UPF2, and UPF3 with full-length MOV10, a modified co-IP strategy was employed. Flag-tagged full-length (fl) UPF1 and MOV10 proteins expressed in HEK293 cells were used as baits and recombinantly purified UPF1, UPF2, and UPF3 were used as preys. Untransfected HEK293 extract was used as a negative control while UPF1fl served as a positive control. For expression, 10 μg plasmid encoding Flag-UPF1fl or Flag-MOV10fl was transfected using polyethyleneimine (Polysciences Inc) into HEK293 cells cultured in 10 cm plates in DMEM high glucose medium (Biowest) supplemented with 10% fetal bovine serum (FBS, Bio&Sell). Cells were harvested 24 h post transfection and lysed in NET-G buffer (50 mM Tris–HCl pH 7.5, 150 mM NaCl, 10% Glycerol, and 0.1% NP40). Expression of Flag-tagged UPF1fl and MOV10fl was confirmed by western blotting using an Anti-Flag (M2) antibody. Approximately 300 μg of lysate was obtained each time, 10% of which was removed to be analyzed as input and the rest used for immunoprecipitation. Flag-UPF1fl and Flag-MOV10fl were immunopurified from cell extracts using 12 μl of Anti-Flag M2 affinity agarose. In addition, 4 to 8 μg of purified UPF1, UPF2, or UPF3 proteins were added to the immobilized Flag-tagged proteins (UPF1fl or MOV10fl) and incubated for 45 min. Beads were washed again with co-IP buffer (20 mM Hepes pH 7.5, 70 mM NaCl, 10% Glycerol, 1 mM MgCl_2_, and 1 μM ZnCl_2_) and the captured proteins were eluted in 50 μl of 1X SDS-PAGE sample loading buffer lacking DTT. Ten micrograms of cell lysate and 0.5 μg of purified proteins were analyzed as input. All immunopurification and coprecipitation experiments were performed in the absence and presence of RNase A (at a final concentration of 60 μg/uL for 10 min at 25 °C) to capture RNA-dependent and RNA-independent interactions mediated by the helicases. Inputs and eluates were analyzed by SDS-PAGE and Coomassie staining. To verify the identity of the protein on SDS-PAGE, the band was excised from the gel and analyzed by peptide-finger printing mass spectrometry.

To determine the UPF2-interacting region of MOV10, Flag-tagged plasmids expressing the NTD (residues 1–264) and the helicase core (residues 289–1001) of MOV10 were generated. Flag-MOV10_NTD_ and Flag-MOV10_hel_ were expressed in HEK293 cells as described for Flag-MOV10fl, which was used as a positive control. Expression of the MOV10 proteins was verified by western Blotting using an anti-Flag antibody. Cell lysates were treated with RNase A and immunoprecipitation and coprecipitation experiments were performed as described above. Inputs and eluates were analyzed on a 10% SDS-PAGE gel and visualized by Coomassie staining. Quantification of UPF2 levels gels was done by performing a densitometric analysis of the bands using ImageJ. The intensity of the UPF2 band in the blank samples (-Flag) was considered as background and subtracted from the intensities of UPF2 bands in all samples with a helicase bait.

## Cell culture for imaging

Human U2OS (American Type Culture Collection) were maintained in low glucose Dulbecco's modified Eagle's medium (DMEM; 01–050A, Sartorius), supplemented with 10% FBS (HyClone Laboratories), 1% Glutamine (Biological Industries) and 1% Penicillin-Streptomycin solution (Biological Industries). For splicing inhibition, cells were treated with pladienolide B (PLB; 0.5 μM, Santa Cruz, 445493) for 6 or 24 h, or with Isoginkgetin (50–100 μM, EMD Millipore, purchased from Sigma-Aldrich, 416154) for 1.5 h before RNA transfection. All sodium arsenite treatments (0.5 mM, Sigma-Aldrich, S7400) were applied 50 min before fixation.

For mRNA transfections, cells were transfected with 1 μg mRNA using Lipofectamine 2000 (Invitrogen, 11668) and fixed after 1.5 h. For transient transfections, cells were transfected with 1 μg of plasmid DNA and 40 μg of sheared salmon sperm DNA (Sigma-Aldrich) when using electroporation (Gene Pulser Xcell, Bio-Rad). Cells were transfected with MOV10-mCherry, UPF1-GFP, MOV10_NTD_-UPF1_hel_-GFP, MOV10 K530A-RFP, GFP-DCP1a, and TIA1-Cherry.

## Immunofluorescence

Cells were grown on coverslips in a 12-well plate and fixed in 4% paraformaldehyde for 20 min. Cells were permeabilized in 0.5% Triton X-100 for 2 min, and blocking was applied using 5% BSA fraction V (MP Biomedicals, 160069). Then, cells were incubated with primary antibodies for 1 h, washed with 1×PBS, and incubated with secondary fluorescent antibodies. The nucleus was stained with Hoechst H33342 (1 μg/ml, Sigma-Aldrich, B2261) and coverslips were mounted in mounting medium (homemade). Primary antibodies: anti-MOV10 (1:350, Abcam, Ab80613), anti-UPF1 (1:350, Abcam, ab109363), anti-HEDLS/p-p70 S6 kinase (1:200, Santa Cruz, sc-8416), anti-G3BP1 (1:400, Abcam, ab56574), anti-Caprin (1:400, Abcam, ab205377), anti-Dcp1a (1:350, Abcam, ab183709), and FMRP (1:350, Abcam, ab17722). Secondary antibodies were purchased from Abcam [Alexa Fluor 488 goat anti-mouse (1:1000, ab150113), Alexa Fluor 488 goat anti-rabbit (1:1000, ab150077), Cy7-Alexa Fluor goat anti-mouse (1:500, ab175738) and Cy5 donkey anti-goat (1:1000, ab6566)].

## Imaging

Wide-field fluorescence images were obtained using the cellSens system based on an Olympus IX83 fully motorized inverted microscope (60 × UPlanXApo objective, 1.42 NA) fitted with a Prime BSI sCMOS (Teledyne) driven by the cellSens software. Colocalization analysis of two channels was performed using ImageJ. Statistical analysis and figure preparation was done using Graphpad Prism 10. Live-cell imaging was carried out using the cellSens system with rapid wavelength switching. Cells were plated on glass-bottomed tissue culture plates (MatTek) in medium containing 10% FBS. Imaging was carried out at 37 °C, using an incubator that includes temperature and CO_2_ control (Life Imaging Services). Live-cell movies were edited by ImageJ.

## Data availability

All original data will be made available upon request.

## Supporting information

This article contains supporting information (54).

## Conflict of interest

The authors declare that they have no conflicts of interest with the contents of this article.
