# Supplementary material for: Functional investigation of the RNA helicase MOV10 with respect to its interplay with factors involved in nonsense-mediated mRNA decay
Source: J Biol Chem. 2025 Jun 24;301(8):110418. doi: 10.1016/j.jbc.2025.110418 (PMC12305709; doi:10.1016/j.jbc.2025.110418)
Supplement: Supplementary Data [file mmc1.pdf]

Supplemental Information

Functional investigation of the RNA helicase MOV10 with respect to its interplay with factors involved in nonsense-mediated mRNA decay

Guangpu Xue, Gabriel P. Faber, Lea S. Pommerening, Megha Mallick, Aditi Gupta, Markus C. Wahl, Yaron Shav-Tal and Sutapa Chakrabarti

A

```
MOV10-NTD_Human    1 MPSKFSCRQLREAGQCFESFLVVRGLDMETDRERLRTIYNRDFKISFGTPAPGFSSMLYG
UPF1-NTD_Human     1 -----MSVEAYGPSSQTLTFLDTEEAELLGADTQGSEFEFTDFTLPSQTQTPPGGPGGPG
                           . :   ...   :** ..  ::     :   :   **.:.  * :*  ..   *

MOV10-NTD_Human   61 MKIANLAYVTKTRVRFFRLDRWADVRFPEKRRMKLGSDISKHHKSLLAKIFYDRAEYLHG
UPF1-NTD_Human    56 GGGAGSPGGAGAGAAAGQLDAQVGPEG-ILQNGAVDDSVAKTSQLLAELNFEEDEEDTYY
                        *. .  : : .   :**  .. .    :.  :...::*  : *    * :  *  :

MOV10-NTD_Human  121 KHGVDVEVQGPHEARDGQLLIRLDLNRKEVLTLRLRNGGTQSVTLTHLFPLCRTPQFAFY
UPF1-NTD_Human   115 TKDLPIHACSYCGIHDPACVVYCNTSKKWFCNGRGNTSGSHIVNH-LVRAKCKEVTLHKD
                     .:.: :.. .    :*   ::  : .:* . . * ...*:: *.   : . *:   :

MOV10-NTD_Human  181 NEDQELPCPLGPGECYELHVH--------CKTSFVGYFPATVLWELLGPGESGSEGAGTF
UPF1-NTD_Human   174 GPLGETVLECYNCGCRNVFLLGFIPAKADSVVVLLCRQPCASQSSLKDINWDSSQWQPLI
                     .   *         * ::.:         . . ::   *.:   .* . . ..*:    :

MOV10-NTD_Human  233 YIARFLAAVAHSPLAAQLKPMTPFKRTRITGN------- 264
UPF1-NTD_Human   234 QDRCFLSWLVKIPSEQEQLRARQITAQQINKLEELWKEN 272
                         **: :.: *   :      :.  :*.
```

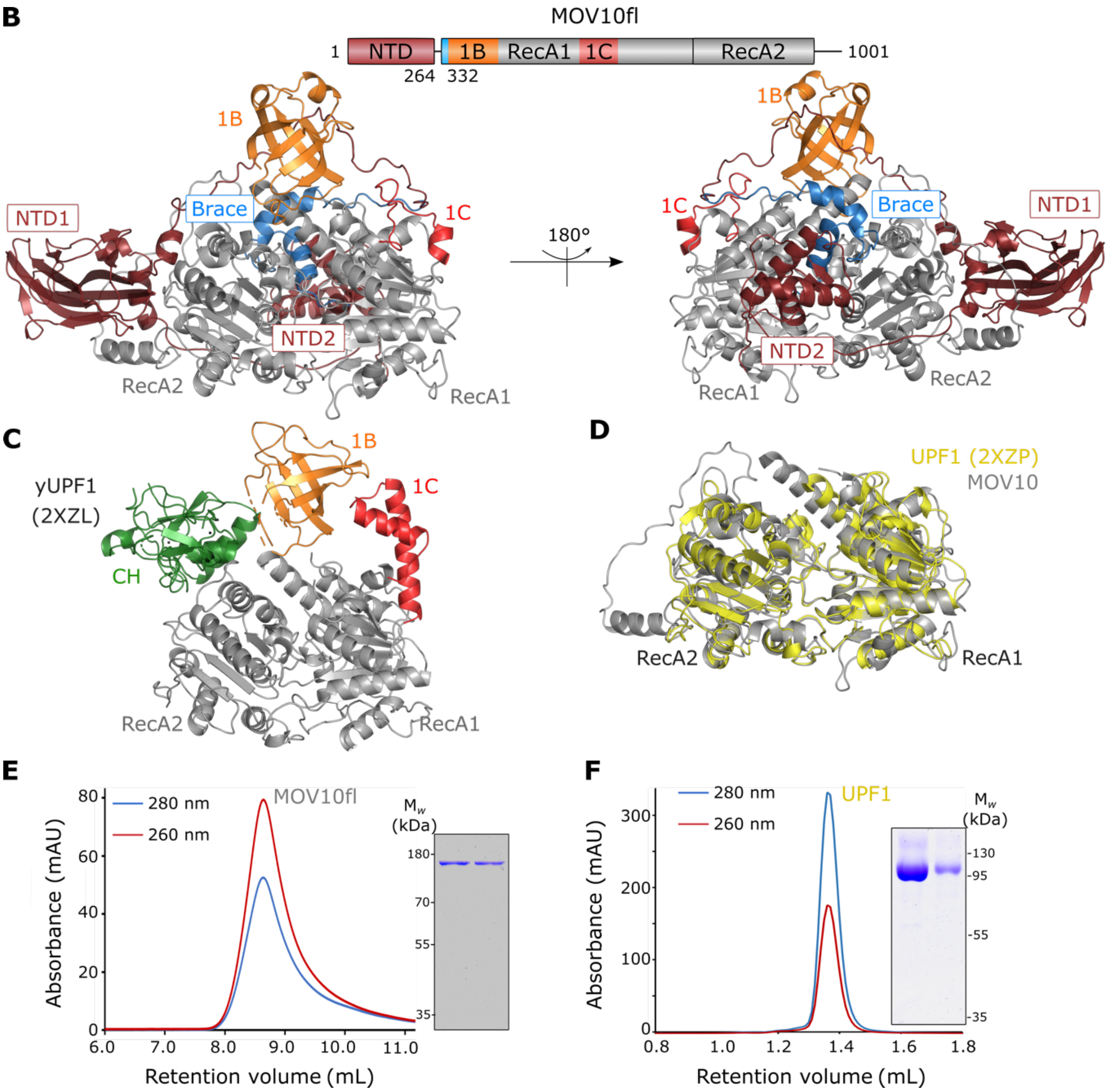

Supplementary figure 1. Structural similarities and differences between SF1 helicases MOV10 and UPF1

A. Sequence alignment of the N-terminal domains (NTD) of human MOV10 (residues 1-264) and human UPF1 (1-272), generated using CLUSTALW. Identical residues are denoted by ***** and conserved and semi-conserved substitutions are denoted by **:** and **.**, respectively. The primary structure of the CH domain of UPF1 (residues 115-272) is indicated by a light green box, and the three zinc finger motifs therein highlighted in red, green and magenta. Sequence motifs typical of zinc fingers are absent in the MOV10-NTD, suggesting that it adopts a fold distinct from the UPF1 CH domain.

B. Domain organization and Alphafold2-predicted structural model of full-length human MOV10. The domains are colored as follows: NTD – brown, the "brace" helices – blue, the RecA1 and RecA2 domains that make up the helicase core – grey, 1B – orange, 1C – red. The NTD can be divided in 2 distinct modules – NTD1, which consists of a β-sheet flanked by α-helices on one side and NTD2, a 3-helix bundle capped by a β-hairpin. The two modules are connected by a long linker, suggesting a flexible orientation of the two modules relative to one another.

C. X-ray crystal structure of the yeast UPF1 (2XZL), showing the N-terminal CH domain (in green). All other domains of the helicase are colored as in **B** above. This arrangement of the CH domain relative to the helicase core represents the "closed" conformation that is achieved upon RNA binding. In the absence of RNA or in the UPF2-bound form, UPF1 adopts as "open" conformation where the CH domain is positioned in proximity to the RecA1 domain (Clerici *et al.*, 2009 and Langer *et al.*, 2024, refs. 20 and 40 of main text, respectively).

D. Structural superposition of the helicase core of human UPF1 (yellow) and human MOV10 (grey). The NTD of MOV10 and the auxiliary domains of both helicases have been omitted for clarity. The RecA domains superpose with an r.m.s.d. of ~2 Å over 94 % of the Cα atoms, indicating an overall high structural similarity between the helicase core domains.

E-F. SEC and corresponding SDS-PAGE analysis of full-length MOV10, expressed using a baculovirus expression system (E) and UPF1, expressed in *E. coli* (F). The MOV10fl obtained after a 3-step purification is free of protein contaminants but associated with nucleic acid and is mostly aggregated whereas UPF1 is free of any protein or nucleic acid contaminants.

```
MOV10hel_Human    332 ---KWRNYEVKLRLLLHLEELQMEHDIRHYDLESVPMTWDPVDQNPRLLTLEVPGVTESR
UPF1hel_Human     295 RYEDAYQYQNIFGPLVKLEADYDKKLKESQTQDNITVRWDLGLNKKRIAYFTLPKTDSDM
                            :*:  :   *::**    ::  .    :.:.: **    :: *:   : :* . ..

MOV10hel_Human    389 PSVLRGDHLFALLSSETHQEDPITYKGFVHKVELDRVKLSFSMSLLSRFVDGLTFKVNFT
UPF1hel_Human     355 RLMQGDEICLRYKGDLAPLWKGIGHVIKVPDNYGDEIAIELRSSVGAPVEVTHNFQVDFV
                        :  .:  :    .. :   . * :    *  .   *.: :.:   *: :  .    .*:*:*.

MOV10hel_Human    449 FNRQPLRVQHRALELTGRWLLWPMLFPVAPRDVPLLPSDVKLKLYDRSLESNPE------
UPF1hel_Human     415 WKSTSFDRMQSALKTFAVDETSVSGYIYHKLLGHEVEDVITKCQLPKRFTAQGLPDLNHS
                      :: .:   : **:  .         :         : . :.     : : ::   *:  ::

MOV10hel_Human    503 QLQAMRHIVTGTTRPAPYIIFGPPGTGKTVTLVEAIKQVVKHLPKAHILACAPSNSGADL
UPF1hel_Human     475 QV----YAVKTVLQRPLSLIQGPPGTGKTVTSATIVYHLARQG-NGPVLVCAPSNIAVDQ
                      *     : *. . : .  :* *********** .  : ::.::  :.  :*.***** ..*

MOV10hel_Human    563 LCQRLRVHLPSSIYRLLAPSRDIRMVPEDIKPCCNWDAKKGEYVFP--------------
UPF1hel_Human     530 LTEKIHQTGLKV-VRLCAKSREAIDSPVSFLALHNQIRNMDSMPELQKLQQLKDETGELS
                      *::::          .**      :.   *             :

MOV10hel_Human    609 -AKKKLQEYRVLI--------------TLITAGRLVSAQFPIDHFTHIFIDEAGHCMEPE
UPF1hel_Human     589 SADEKRYRALKRTAERELLMNADVICCTCVGAGDPRLAKMQ---FRSILIDESTQATEPE
                       *  *                          * :  **     *          *.***      ***

MOV10hel_Human    655 SLVAIAGLMEVKETGDPGGQLVLAGDPRQLGPVLRSPLTQKHGLGYSLLERLLTYNSLYK
UPF1hel_Human     646 CMVPV---------VLGAKQLILVGDHCQLGPVVMCKKAAKAGLSQSLFERLVVLG----
                        * :            . **:*.**  *****: .  : * **. **:***:. .

MOV10hel_Human    715 KGPDGYDPQFITKLLRNYRSHPTILDIPNQLYYEGELQACADVVDRERFCRWAGLPRQGF
UPF1hel_Human     693 --------IRPIRLQVQYRMHPALSAFPSNIFYEGSLQNGVTAADRVKKGFDFQWPQPDK
                                  :*   :** **::  :*.:::***.**   . ..** :       *.

MOV10hel_Human    775 PIIFHGVMGKDEREGNSPSFFNPEEAATVTSYLKLLLAPSSKKGKARLSPRSVGVISPYR
UPF1hel_Human     745 PMFFYVTQGQEEIASSGTSYLNRTEAANVEKITTKLLKAGAK-------PDQIGIITPYE
                      *::*    * :*   ....*::*  ***.*  .   *** ..:*        *  :*:*:**

MOV10hel_Human    835 KQVEKIRYCITKLDRELRGLDDIKDLKVGSVEEFQGQERSVILISTVRSSQSFVQLDLDF
UPF1hel_Human     798 GQRSYLVQYMQFSG-SLHTKLYQ-EVEIASVDAFQGREKDFIILSCVRAN-------EHQ
                       *      :   .      .     :: ::**: *** *:..*::* **:.        .

MOV10hel_Human    895 NLGFLKNPKRFNVAVTRAKALLIIVGNPLLLGHDPDWKVFLEFCKENGGYTGCPFPAKLD
UPF1hel_Human     849 GIGFLNDPRRLNVALTRARYGVIIVGNPKALSKQPLWNHLLNYYKEQKVLVEGPLNNLRE
                      .:***::*:*:***:***:  :****** *.::* *: :*:: **    .    *.    :

MOV10hel_Human    955 LQQGQNLLQGLSKLSPSTSGPHSHDYLPQEREGEGGLSLQVEPEWRNEL 1003
UPF1hel_Human     909 SLMQFS------------------------------------------- 914
```

Supplementary figure 2. Sequence similarity between the MOV10 and UPF1 helicase cores

Sequence alignment of the helicase core of human MOV10 (residues 332-1001) and human UPF1 (295-914), generated using CLUSTALW. Sequence conservation is denoted as described in Supplementary figure 1A. The conserved helicase motifs, as described in Fairman-Williams *et al.*, 2010 (ref. 8 of main text), are colored by function: red – ATP binding and hydrolysis, blue – RNA binding, yellow – coordination between ATP and RNA binding sites. The invariant Walker A and Walker B motifs present in all NTPase are highlighted by a red box.

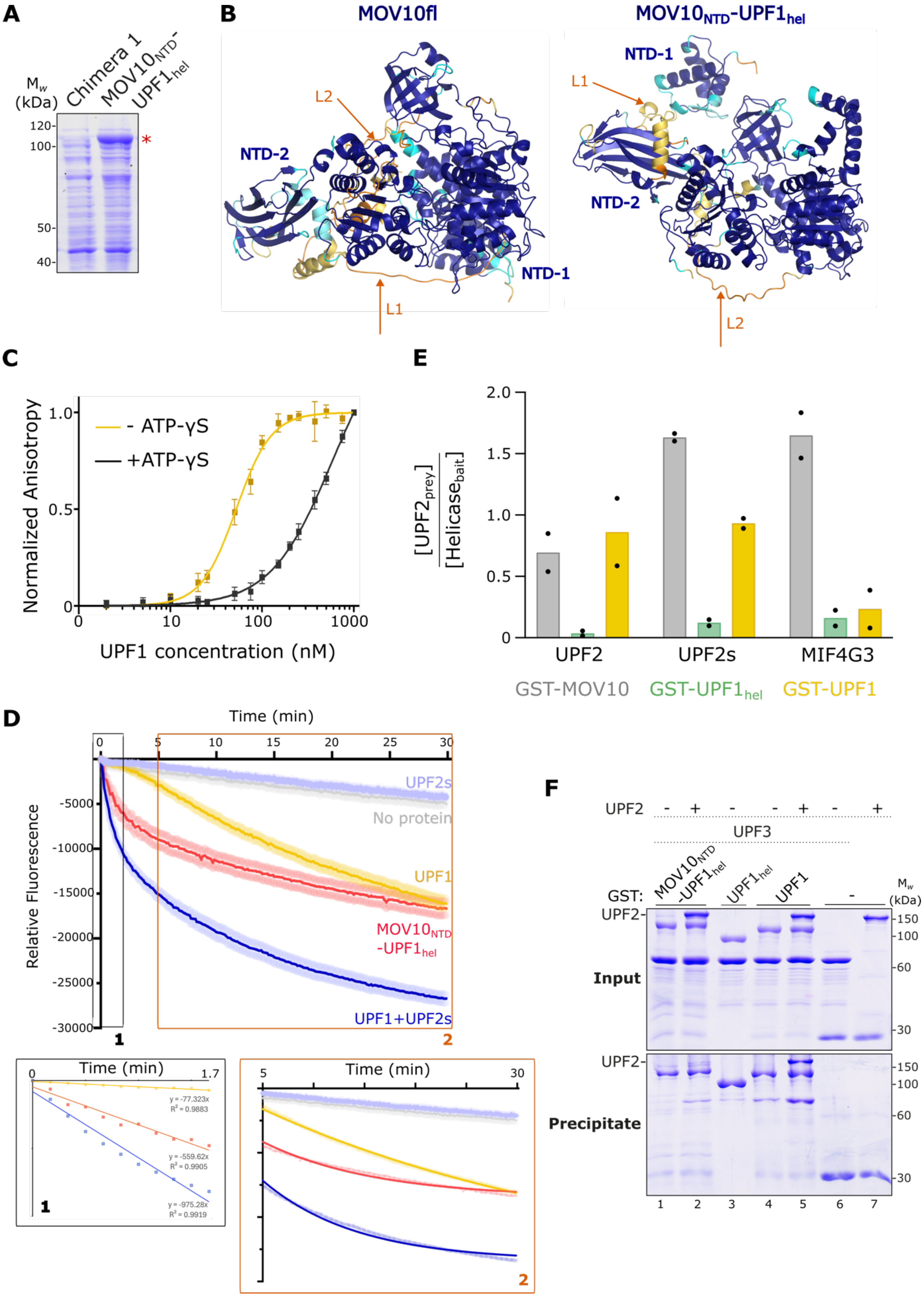

A
Mw (kDa)
Chimera 1
MOV10NTD-UPF1hel
120
100
50
40
*
B
MOV10fl
MOV10NTD-UPF1hel
NTD-1
NTD-2
L1
L2
C
Normalized Anisotropy
- ATP-γS
+ATP-γS
1.0
0.5
0
0
10
100
1000
UPF1 concentration (nM)
D
Time (min)
0
5
10
15
20
25
30
Relative Fluorescence
-5000
-10000
-15000
-20000
-25000
-30000
UPF2s
No protein
UPF1
MOV10NTD-UPF1hel
UPF1+UPF2s
1
2
Time (min)
0
1.7
y = -77.323x
R² = 0.9883
y = -559.62x
R² = 0.9905
y = -975.28x
R² = 0.9919
1
Time (min)
5
30
2
E
[UPF2prey]/[Helicasebait]
2.0
1.5
1.0
0.5
0
UPF2
UPF2s
MIF4G3
GST-MOV10
GST-UPF1hel
GST-UPF1
F
UPF2
- + - - + - +
UPF3
GST:
MOV10NTD-UPF1hel
UPF1hel
UPF1
-
Mw (kDa)
UPF2-
150
100
60
30
Input
Precipitate
1
2
3
4
5
6
7

Supplementary figure 3. Characterization of the MOV10-UPF1 chimeric protein

A. SDS-PAGE analysis of the crude soluble lysate of Chimera 1 and $MOV10_{NTD}$-$UPF1_{hel}$ (Chimera 3). Inclusion of the brace helices renders Chimera 1 insoluble, and therefore unsuitable for biochemical studies.

B. Alphafold predicted structural models of MOV10fl and $MOV10_{NTD}$-$UPF1_{hel}$. The structures are coloured according to the per-residue confidence score (pLDDT) of prediction: dark blue – very high (pLDDT>90), cyan – high (90>pLDDT>70), yellow – low (70>pLDDT>50), orange – very low (pLDDT<50). While the two NTD modules and the helicase core domains are predicted with high confidence for both proteins, the linker connected NTD-1 and NTD-2 (L1) and the NTD to the helicase core (L2) are predicted with very low confidence, suggesting a high degree of structural flexibility.

C. Quantitative measurement of RNA-binding affinities of UPF1, in the absence (yellow trace) and presence of a non-hydrolysable ATP analogue ATPγS (black trace) using fluorescence anisotropy. Data representation and curve-fitting is as described for Figure 1C. The affinity of UPF1 for RNA decreases significantly in the presence of ATP analogs (also reported in Chakrabarti *et al.*, 2011 and Gowravaram *et al.*, 2018, refs. 18 and 26 of main text, respectively).

D. Measurement of nucleic acid-unwinding activity of $MOV10_{NTD}$-$UPF1_{hel}$ in comparison to UPF1. The insets 1 and 2 show an enlarged view of the data points for the indicated time range (0-1.7 minutes for inset 1 and 5-30 minutes for inset 2). Data points within inset 1 (denoted by filled squares) were fit to a straight line using linear regression, and the slopes of the lines were compared to determine the relative initial nucleic acid-unwinding activities of the helicases. Data points within inset 2 were fit to a one-step exponential decay model using Prism 10, indicated by solid lines. The decay rate constants were compared to determine the relative overall unwinding activities of the helicases. Error bars associated with each data point have been omitted in the insets for clarity.

E. Quantification of the amounts of UPF2 variants co-precipitated with GST- $MOV10_{NTD}$-$UPF1_{hel}$, GST-$UPF1_{hel}$ and GST-UPF1 in figure 2E of the main text. Densitometric analysis of the bands corresponding to helicase baits and UPF2 variants was done using ImageJ. The amounts of UPF2 co-precipitated in each sample were normalized to the amount of helicase

bait in the same sample. The columns represent the mean of two independent experiments; individual data points are shown as black circles.

F. GST-pulldown assay to test the interaction of $MOV10_{NTD}$-$UPF1_{hel}$ with UPF3, in the absence and presence of UPF2. GST- $MOV10_{NTD}$-$UPF1_{hel}$ was used as a bait and GST-UPF1 served as a positive control. GST and GST-$UPF1_{hel}$ were used as negative controls. No direct interaction between $MOV10_{NTD}$-$UPF1_{hel}$ and UPF3 was observed. $MOV10_{NTD}$-$UPF1_{hel}$ engages UPF3 only in the presence of UPF2. GST-UPF1 showed a weak binding to UPF3 alone, and a stronger binding in the presence of UPF2, as reported in previous studies (Chamieh *et al.*, 2008, ref. 19 of main text). No significant interaction with UPF3 was detected using the helicase core of UPF1 ($UPF1_{hel}$) as a bait. The top and bottom panels show the input and precipitate, respectively.

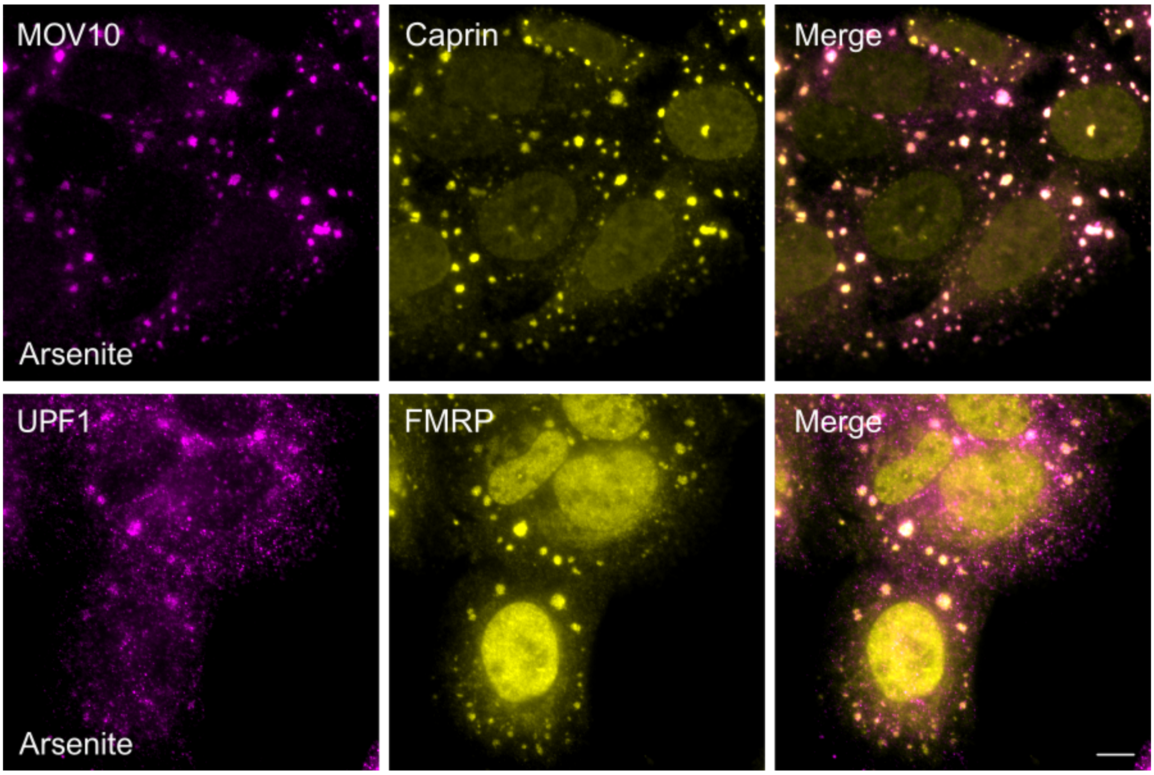

Supplementary figure 4. MOV10 and UPF1 are recruited to SGs after arsenite exposure

U2OS cells were treated with arsenite for 1 h, and cells were stained with anti-caprin or anti-FMRP to mark SGs (yellow) and either anti-MOV10 antibody (top panel) or anti-UPF1 antibody (bottom panel, magenta). Both proteins localize to stress granules, and indeed the bodies formed (magenta) are canonical SGs.

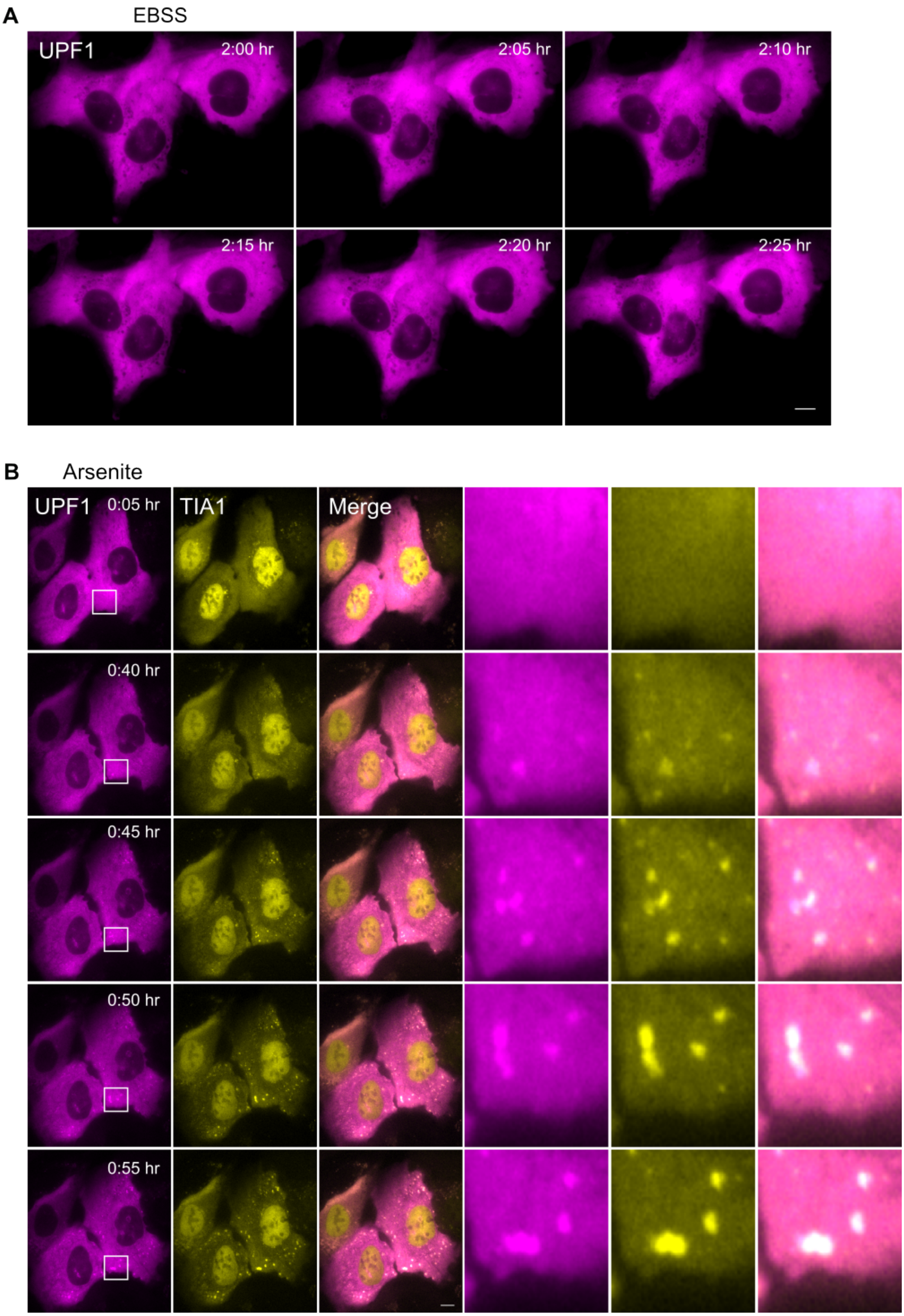
A
EBSS
UPF1
2:00 hr
2:05 hr
2:10 hr
2:15 hr
2:20 hr
2:25 hr
B
Arsenite
UPF1
0:05 hr
TIA1
Merge
0:40 hr
0:45 hr
0:50 hr
0:55 hr

Supplementary figure 5. Live cell imaging of recruitment of UPF1 to cytoplasmic bodies

A. Frames from time-lapse movies showing PBs in U2OS cells expressing UPF1-GFP after 2 hr of incubation in EBSS medium. UPF1 remains diffuse throughout the cytoplasm and does not enter PBs.

B. Frames from time-lapse movies of U2OS cells expressing TIA1-RFP and UPF1-GFP under treatment with arsenite (0.5 mM). As SGs begin to form, UPF1 can be seen aggregating at newly formed granules. Scale bars = 10 μm.

A

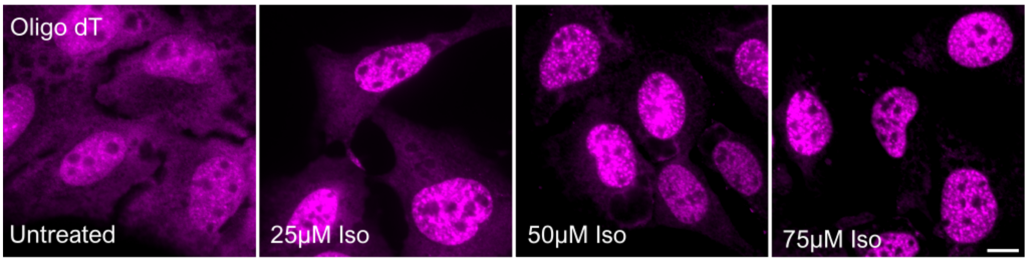

B

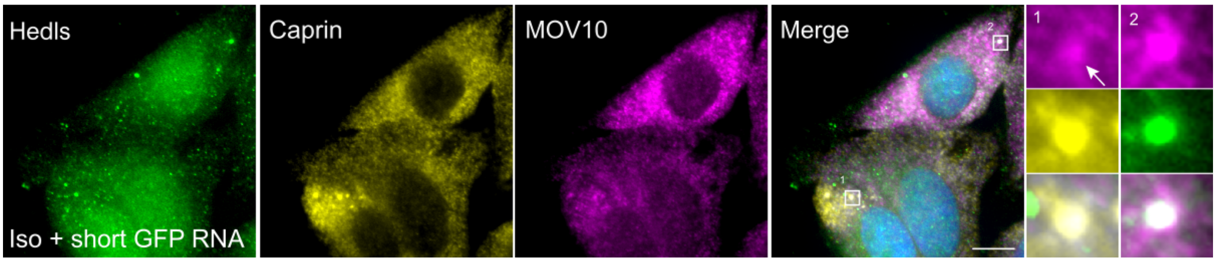

Supplementary figure 6. RNA depleted cells are still capable of forming PBs

A. U2OS cells were treated with different amounts of the splicing inhibitor isoginkgetin (Iso) for 5 h and stained by RNA FISH using a fluorescent probe to poly(A)$^{+}$ RNA. Increasing amounts of isoginkgetin prevents RNA export to the cytoplasm and alters nuclear bodies. Scale bars = 10 µm.

B. U2OS cells were treated with isoginkgetin (Iso, 50 µM) for 5 h overall and transfected with the short GFP RNA (0.75–1 µg) for 1.5 h before fixation. Cells were stained with anti-Caprin to mark SGs (yellow) and anti-Hedls to mark PBs (green) as well as anti-MOV10. The merged image shows that all cells, even those that did not form SGs after RNA transfection, contained PBs. MOV10 can be found both in SGs after formation (box 1) and in PBs in cells that did not receive RNA (box 2). DNA Hoechst staining is shown in blue. Scale bars = 10 µm.

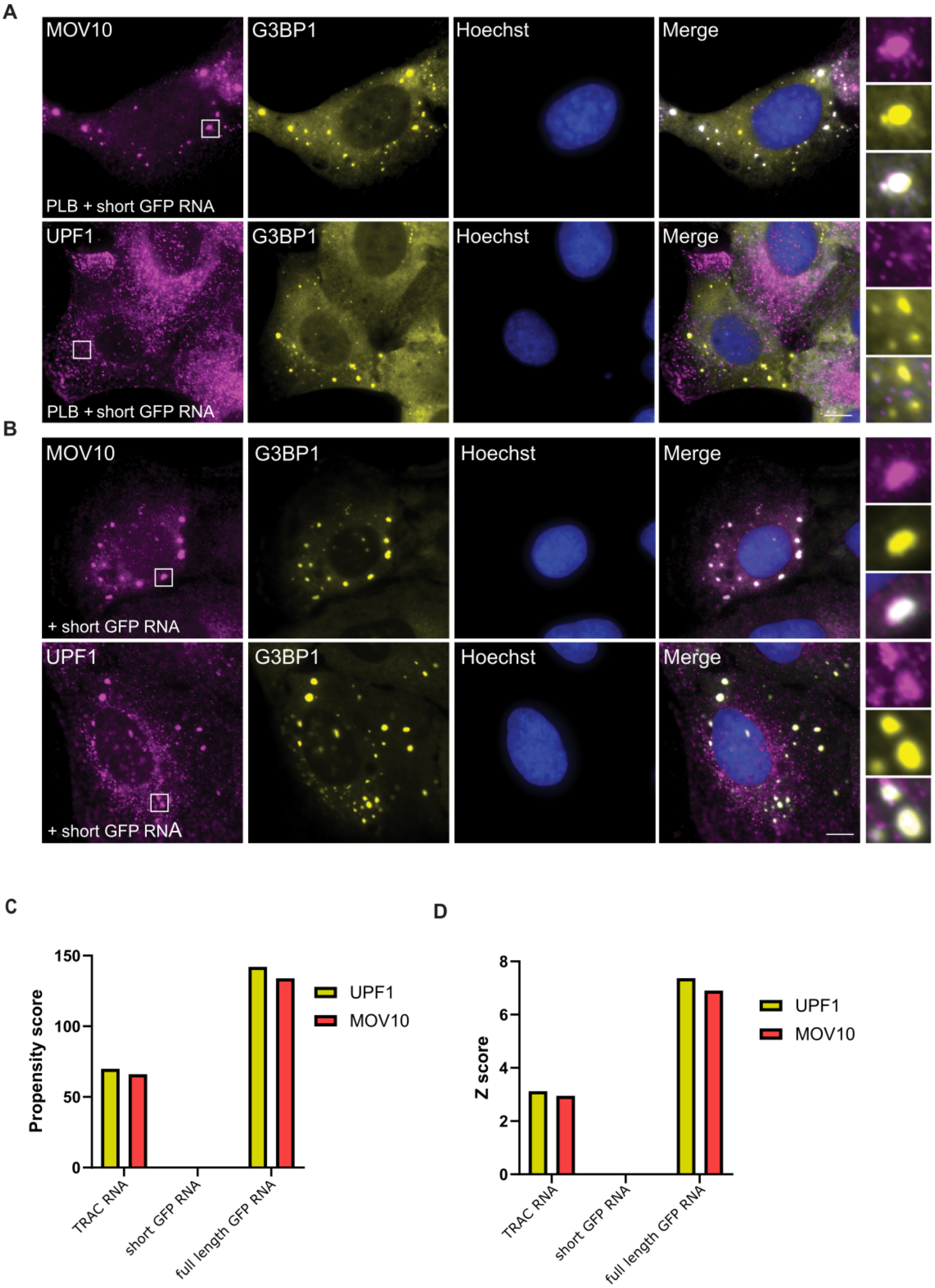

Supplementary figure 7. UPF1 is unable to localize to SGs following mRNA depletion after splicing inhibition

A. U2OS cells were treated with PLB (0.5 µM) for 24 hrs. overall and transfected with short GFP RNA (1 µg), using Lipofectamine 2000, and fixed after 1.5 h. Cells were stained using anti-G3BP1 (yellow), and either MOV10 in top panel or UPF1 in bottom panel (magenta). As seen when with isoginkgetin, while MOV10 localized to the SGs, UPF1 did not. DNA Hoechst staining is shown in blue. Scale bars = 10 µm.

B. U2OS cells were transfected with short GFP RNA (1 µg) and fixed after 1.5 h. Cells were stained for G3BP1 (yellow) and MOV10 or UPF1 (purple) as described for Figure 7. Both MOV10 and UPF1 successfully localized to SGs in the presence of cellular mRNA. Scale bars = 10 µm.

C-D. Prediction scores of UPF1 and MOV10 interaction with synthetic mRNAs. Both MOV10 and UPF1 showed high scoring hits with TRAC RNA and full length GFP RNA, but not with the short GFP RNA.

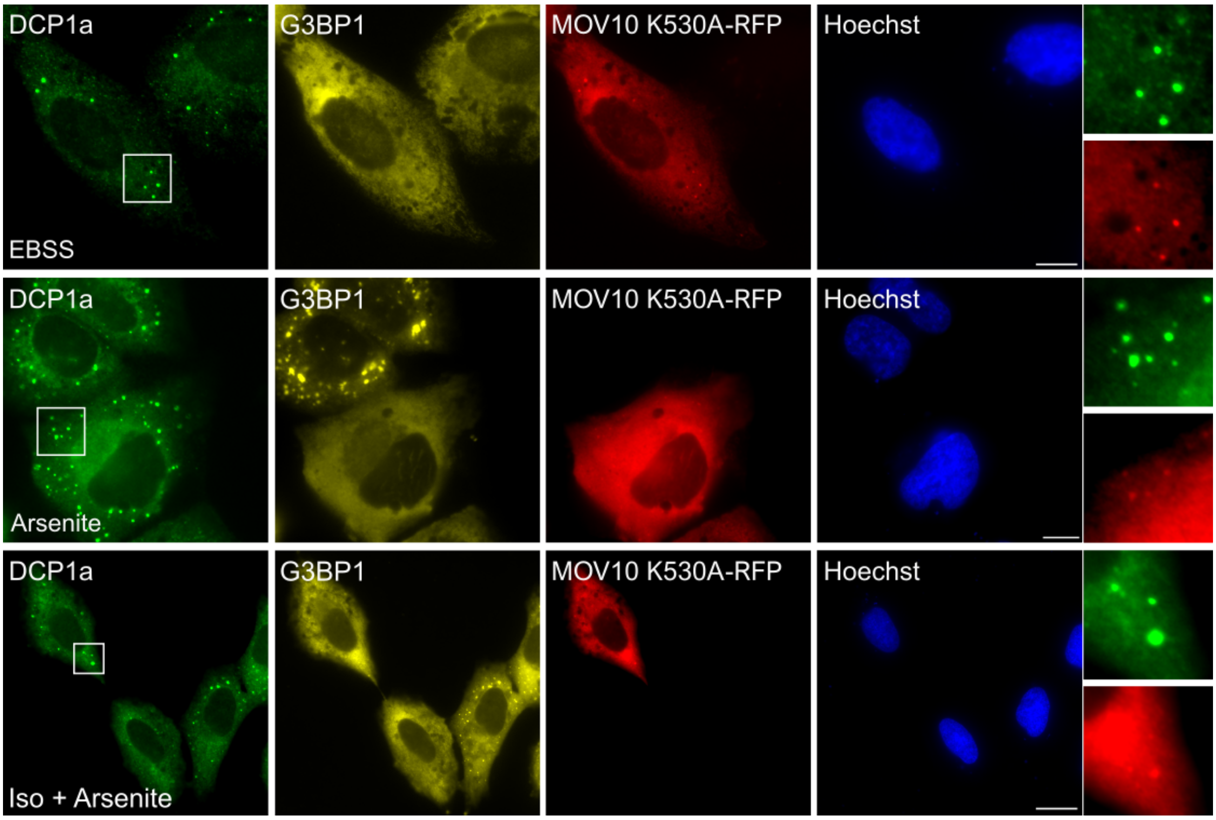

Supplementary figure 8. A catalytically inactive variant of MOV10 shows altered localization to cytoplasmic bodies.

U2OS cells were transfected with MOV10 K530A-RFP and stained with anti-G3BP1 to mark SGs (yellow) and anti-Hedls to mark PBs (green). Mutant MOV10 protein is still capable of localizing to PBs but does not localize to SGs. DNA Hoechst staining is shown in blue. Scale bars = 10 µm.

Supplementary Table 1: Expression plasmids of human MOV10 variants

Vector	Constructs	Description	Soluble
pEC-K-3C-GST	MOV10 full-length	*E. coli* expression, NT-His-GST tag	No
pEC-K-3C-Trx	MOV10 full-length	*E. coli* expression, NT-His-Trx tag	No
pEC-K-3C-His	MOV10 full-length	*E. coli* expression, NT-His tag	No
pEC-A-HT-His	MOV10 full-length	*E. coli* expression, CT-His tag	No
pEC-K-3C-MBP	MOV10 full-length	*E. coli* expression, NT-His-MBP tag	No
pT7-His-NT*-QG	MOV10 full-length	*E. coli* expression, NT-His-NT* tag	No
pEC-A-3C-Trx	[¶] UPF1(115-272)-Linker-MOV10(298-967)	Fusion of the UPF1-CH domain with the MOV10 brace-helicase domains. [#]Linker: GSAGAAGSGA, NT-His-Trx tag	No
pEC-A-3C-Trx	UPF1(115-272)-Linker-MOV10(332-967)	Fusion of the UPF1-CH domain with the MOV10 helicase core, NT-His-Trx tag	No
pEC-A-3C-Trx	MOV10(1-251)-Linker-UPF1(295-914)	Fusion of the MOV10-NTD with the UPF1 helicase core, NT-His-Trx tag	Yes
pEC-A-3C-His	MOV10(1-251)-Linker-UPF1(295-914)	Fusion of the MOV10-NTD with the UPF1 helicase core, NT-His tag	Yes
pEC-A-3C-Trx	MOV10(1-264)-Linker-UPF1(295-914)	Fusion of the MOV10-NTD with the UPF1 helicase core, NT-His-Trx tag	Yes
pEC-A-3C-His	MOV10(1-264)-Linker-UPF1(295-914)	Fusion of the MOV10-NTD with the UPF1 helicase core, NT-His tag	Yes
pEC-K-3C-GST	MOV10(1-264)-Linker-UPF1(295-914)	Fusion of the MOV10-NTD with the UPF1 helicase core, NT-His-GST tag	Yes
pETMCN-A-His-TEV	MOV10(1-332)-GSAG-UPF1(295-914)	Fusion of the MOV10-NTD-brace region with the UPF1 helicase core, separated by a GSAG linker, NT-His tag	No

[¶] All chimeric proteins were generated using human UPF1.
[#] The linker connecting the UPF1 and MOV10 domains is the same in all chimeric proteins, unless specified.

Supplementary Table 2: Identification of UPF2 by MALDI-TOF-MS.

Start – End	Observed	Mr(expt)	Mr(calc)	ppm	M	Peptide
67 – 78	1267.7022	1266.6949	1266.6568	30.1	0	K.LNIDQNPGTAPK.Y
130 – 144	1911.9658	1910.9585	1910.9122	24.2	1	K.EKEESIQLHQEAWER.H
132 – 144	1654.8192	1653.8120	1653.7747	22.6	0	K.EESIQLHQEAWER.H
155 – 170	1907.9104	1906.9031	1906.8558	24.8	0	K.NQNAPDSRPEENFFSR.L
194 – 207	1546.7866	1545.7793	1545.7423	23.9	0	R.DSLSHDFNGLNLSK.Y
307 – 320	1657.8428	1656.8355	1656.8042	18.9	0	R.ESHTHVSVVISFCR.H
614 – 620	815.5468	814.5396	814.5065	40.6	0	R.ALFIVPR.Q
623 – 631	1107.6257	1106.6184	1106.6124	5.45	0	R.LDLLPFYAR.L
653 – 660	1033.5527	1032.5455	1032.5254	19.5	1	R.GDFRFHVR.K
724 – 730	825.4543	824.4470	824.4140	39.9	0	R.SPESHLR.T
775 – 783	1157.6789	1156.6716	1156.6353	31.4	0	K.RPPLQEYVR.K
775 – 784	1285.7769	1284.7696	1284.7302	30.6	1	K.RPPLQEYVRK.L
881 – 889	1174.6278	1173.6206	1173.5818	33.0	0	K.FLGELYNYR.M
890 – 898	1067.5931	1066.5859	1066.5481	35.4	0	R.MVESAVIFR.T + Oxidation (M)
996 – 1005	1220.6311	1219.6238	1219.5867	30.5	0	K.LCNSLEESIR.Q
1127 – 1135	1194.6042	1193.5969	1193.5533	36.6	0	K.MMLENLQQR.S + 2 Oxidation (M)
1127 – 1141	1765.7776	1764.7703	1764.8498	-45.1	1	K.MMLENLQQRSGESVK.V + Oxidation (M)
1222 – 1229	973.5650	972.5577	972.5240	34.7	0	K.LTLDINER.Q

1 MHHHHHHGMS DKIIHLTDDS FDTDVLKADG AILVDFWAEW CGPCKMIAPI

51 LDEIADEYQG KLTVAK**LNID** **QNPGTAPK**YG IRGIPTLLLF KNGEVAATKV

101 GALSKGQLKE FLDANLTSLE VLFQGPDMK**E** **KEESIQLHQE** **AWER**HHLRKE

151 LRSK**NQNAPD** **SRPEENFFSR** LDSSLKKNTA FVKKLKTITE QQR**DSLSHDF**

201 **NGLNLSK**YIA EAVASIVEAK LKISDVNCAV HLCSLFHQRY ADFAPSLLQV

251 WKKHFEARKE EKTPNITKLR TDLRFIAELT IVGIFTDKEG LSLIYEQLKN

301 IINADR**ESHT** **HVSVVISFCR** HCGDDIAGLV PRKVKSAAEK FNLSFPPSEI

351 ISPEKQQPFQ NLLKEYFTSL TKHLKRDHRE LQNTERQNRR ILHSKGELSE

401 DRHKQYEEFA MSYQKLLANS QSLADLLDEN MPDLPQDKPT PEEHGPGIDI

451 FTPGKPGEYD LEGGIWEDED ARNFYENLID LKAFVPAILF KDNEKSCQNK

501 ESNKDDTKEA KESKENKEVS SPDDLELELE NLEINDDTLE LEGGDEAEDL

551 TKKLLDEQEQ EDEEASTGSH LKLIVDAFLQ QLPNCVNRDL IDKAAMDFCM

601 NMNTKANRKK LVR**ALFIVPR** QR**LDLLPFYA** **R**LVATLHPCM SDVAEDLCSM

651 LR**GDFRFHVR** KKDQINIETK NKTVRFIGEL TKFKMFTKND TLHCLKMLLS

701 DFSHHHIEMA CTLLETCGRF LFR**SPESHLR** TSVLLEQMMR KKQAMHLDAR

751 YVTMVENAYY YCNPPPAEKT VKKK**RPPLQE** **YVRK**LLYKDL SKVTTEKVLR

801 QMRKLPWQDQ EVKDYVICCM INIWNVKYNS IHCVANLLAG LVLYQEDVGI

851 HVVDGVLEDI RLGMEVNQPK FNQRRISSAK **FLGELYNYRM** **VESAVIFR**TL

901 YSFTSFGVNP DGSPSSLDPP EHLFRIRLVC TILDTCGQYF DRGSSKRKLD

951 CFLVYFQRYV WWKKSLEVWT KDHPFPIDID YMISDTLELL RPKIK**LCNSL**

1001 **EESIR**QVQDL EREFLIKLGL VNDKDSKDSM TEGENLEEDE EEEEGGAETE

1051 EQSGNESEVN EPEEEEGSDN DDDEGEEEEE ENTDYLTDSN KENETDEENT

1101 EVMIKGGGLK HVPCVEDEDF IQALDK**MMLE** **NLQQRSGESV** **K**VHQLDVAIP

1151 LHLKSQLRKG PPLGGGEGEA ESADTMPFVM LTRKGNKQQF KILNVPMSSQ

1201 LAANHWNQQQ AEQEERMRMK K**LTLDINER**Q EQED

The table shows information for all identified peptides (highlighted in red in the sequence below) that were matched to UPF2. UPF2 was identified with a significant Mascot Score of 60, corresponding to an expect value of 0.019. Observed = measured m/z, Mr(expt) = measured peptide mass (uncharged), Mr(calc) = theoretical peptide mass, ppm = mass deviation in parts per million, M = number of missed cleavages, Peptide = peptide sequence.

Supplementary Methods

The gel band corresponding to UPF2 was excised from the gel and digested into peptides by Trypsin following a protocol described elsewhere (Shevchenko, Tomas et al. 2006). The peptides were applied by the dried-droplet technique using α-cyano-4-hydroxycinnamic acid as a matrix and analyzed by matrix-assisted laser desorption/ionization time of flight mass spectrometry (MALDI-TOF-MS). All measurements were performed with an UltrafleXtreme TOF/TOF instrument (Bruker Daltonics, Bremen, Germany) equipped with a 2000 Hz solid-state Smart beam-II laser. MS spectra (peptide mass fingerprints) were recorded in positive reflector mode over an m/z range of 700–3,500. Data were analyzed using the software FlexAnalysis provided with the instrument. Protein identification was performed by comparison of the resulting peptide mass fingerprint spectra to the human protein sequence database (SwissProt) including the sequence of the UPF2 variant used (human UPF2, residues 121-1227) using the software Mascot (version 2.8.2, Matrix Science, London, UK). The mass tolerance of peptide ions was set to 50 ppm and one missed cleavage site was allowed. Methionine oxidation and acetylation (protein N-terminus) were defined as variable modifications. Carbamidomethylation on cysteines was defined as fixed modification. Protein identifications were based on significance threshold of $p<0.05$ according to the Mascot software.

Reference:

Shevchenko, A., H. Tomas, J. Havlis, J. V. Olsen and M. Mann (2006). "In-gel digestion for mass spectrometric characterization of proteins and proteomes." Nat Protoc **1**(6): 2856-2860.
